# YsxC is a placeholder for ribosomal protein uL2 during 50S ribosomal subunit assembly

**DOI:** 10.1093/nar/gkaf1071

**Published:** 2025-10-28

**Authors:** Amal Seffouh, Dominic Arpin, Kaustuv Basu, Joaquin Ortega

**Affiliations:** Department of Anatomy and Cell Biology, McGill University, 3640 Rue University, Montreal, Quebec H3A 0C7, Canada; Centre de Recherche en Biologie Structurale, McGill University, 3649 Promenade Sir William Osler, Montreal, Quebec H3G 0B1, Canada; Department of Anatomy and Cell Biology, McGill University, 3640 Rue University, Montreal, Quebec H3A 0C7, Canada; Centre de Recherche en Biologie Structurale, McGill University, 3649 Promenade Sir William Osler, Montreal, Quebec H3G 0B1, Canada; Department of Anatomy and Cell Biology, McGill University, 3640 Rue University, Montreal, Quebec H3A 0C7, Canada; Centre de Recherche en Biologie Structurale, McGill University, 3649 Promenade Sir William Osler, Montreal, Quebec H3G 0B1, Canada; Department of Anatomy and Cell Biology, McGill University, 3640 Rue University, Montreal, Quebec H3A 0C7, Canada; Centre de Recherche en Biologie Structurale, McGill University, 3649 Promenade Sir William Osler, Montreal, Quebec H3G 0B1, Canada

## Abstract

The maturation of the functional core of the 50S ribosomal subunit in *Bacillus subtilis* is assisted by assembly factors that enhance the efficiency of the process. Two essential assembly factors, the GTPases RbgA and YphC, bind at or near the functional sites of the 50S subunit to promote the folding of ribosomal RNA helices that play key functional roles. YsxC is another GTPase involved in the maturation of the 50S subunit, whose function remains unknown. We demonstrate that YsxC aids 50S assembly through a drastically different mechanism. YsxC binds in the body of the 44.5S large ribosome assembly intermediate, occupying the site where uL2 binds and controls the timing in the folding of rRNA helices forming the binding site for uL2. It creates a “primordial” binding site that includes six out of the eleven rRNA helices forming the uL2 mature binding site. Once YsxC is released, uL2 binds to this “primordial” binding site, and the remaining helices that stabilize uL2 fold, and the entire region adopts the mature conformation. This role of YsxC functioning as a placeholder factor for ribosomal protein uL2 provides the first example of such a factor’s involvement in the ribosome assembly process in bacteria.

## Graphical Abstract

**Graphical Abstract:**
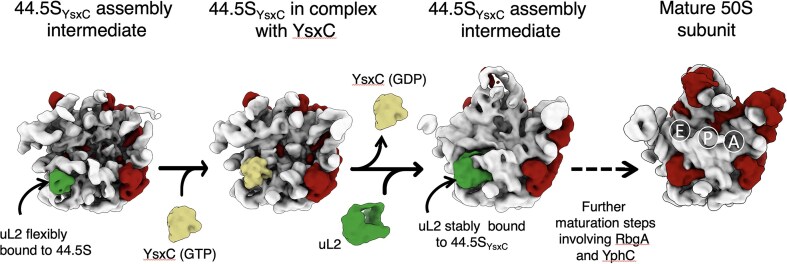

## Introduction

During ribosomal assembly in bacteria, three ribosomal RNA (rRNA) molecules and over 50 ribosomal proteins (r-proteins) come together to form a functional ribosome. An important event in this process is the progressive and co-transcriptional folding of the rRNA molecules. The r-proteins assist in this folding by binding to the rRNA and stabilizing its transient folds as it evolves to its mature conformation [1, 2]. A distinctive feature of the r-proteins is that once they bind to the assembling ribosomal particle, they stabilize transient rRNA folds, remain attached, and become part of the mature ribosome. This characteristic sets r-proteins apart from another group of proteins known as assembly factors. These factors are also involved in the ribosome assembly process; however, unlike r-proteins, they interact with the ribosomal subunits at specific times during assembly and ultimately detach after completing their function.

Some assembly factors enhance the efficiency of rRNA folding, while others introduce chemical modifications at specific nucleotides [3, 4]. Other assembly factors act as checkpoint proteins [5], conducting functional checks in the assembling particle before releasing it to the pool of actively translating ribosomes. Ribosome assembly factors also exist in eukaryotes. In fact, the total number of assembly factors correlates with cellular complexity. Most bacterial species require between 30 and 40 assembly factors [6, 7]. However, in eukaryotes, the increased complexity of the process needs the participation of ∼300 factors in yeast and >600 in human cells [8, 9].

The function of some assembly factors in eukaryotic cells extends beyond the functions described above. Some factors function as “placeholder factors” [10]. In the “classical view,” placeholder factors are paralogous r-proteins that emerged through gene duplication but cannot functionally replace their r-protein counterparts [11, 12]. During assembly, these placeholder factors prevent the premature binding of their r-protein counterparts to their rRNA binding sites, controlling both the timing of their entry and their position in the assembly. In their absence, the corresponding r-proteins are recruited too early to the assembly intermediates, hindering maturation events that only the placeholder factors can facilitate. Examples include Mrt4 and Rlp24 in yeast, which act as placeholder factors for uL10 and eL24 during biogenesis [11, 13]. The exchange between the placeholder and authentic r-protein can occur directly or through an additional factor that facilitates the exchange. This is the case for Yvh1, which is necessary for the exchange of Mrt4 and uL10 [14–16].

Many placeholder factors, such as Rlp7 and lmp3 in yeast, function differently from this classical view and do not compete for the same binding site as their counterpart r-proteins. Rlp7 and lmp3 exhibit considerable sequence homology with r-proteins uL30 and uS4, respectively [17]. However, they bind to a different site than their paralogous r-proteins. It is unclear how proteins with similar structures are targeted to different locations. Current thinking suggests that other cofactors may recruit and divert them to bind at a different site than the r-protein.

There are also placeholder factors whose sequences or structures do not resemble those of r-proteins. However, they can mask important ribosomal functional sites until a specific maturation event has been completed, thereby preventing the recruitment of other assembly factors or r-proteins. An example of this type of placeholder is Tsr1, which blocks the binding of the GTPase elF5B and ATPase Rli1, both of which are required for the complete maturation of the small subunit in yeast [18, 19].

One additional class of placeholder factors mimics the structure of a ribosomal RNA motif typically bound by another RNA motif, thereby delaying the interaction with the “bona fide” interaction partner. Such is the case with the Syo1-HS domain in yeast, which mimics H84 in the 25S rRNA, controlling the timing of uL5’s entry into the 60S assembly intermediate [20]. This placeholder mechanism is known as “protein-RNA mimicry” [13].

Numerous placeholder proteins have been described in yeast and other eukaryotes based on various functional models. However, to our knowledge, no placeholder factors have been identified in the bacterial ribosome assembly process. In this study, we present the first example. YsxC (YihA in *Escherichia coli*) is a 22 kDa essential GTPase in *Bacillus subtilis* [21–23]. Depletion of YsxC in cells leads to the accumulation of a 50S assembly intermediate known as the 44.5S_YsxC_ particle. The 44.5S_YsxC_ contains an identical r-protein complement to the 45S_RbgA_ and 45S_YphC_ particles, which accumulate upon the depletion of RbgA and YphC, two other essential assembly factors involved in the late stages of maturation of the 50S subunit [24–26]. The 45S_RbgA_ and 45S_YphC_ assembly intermediates represent a common assembly intermediate that serves as a convergence node for the multiple parallel pathways forming the folding landscape of the 23S rRNA molecule of the 50S subunit [4]. However, it is still unclear whether the 44.5S_YsxC_ particle is structurally similar to the 45S_RbgA_ and 45S_YphC_ particles or whether it represents an earlier or later maturation step than the 45S particles.

Here, we found that structurally the 44.5S_YsxC_ particle represents an earlier assembly intermediate than the 45S_RbgA_ and 45S_YphC_ particles. We observed that in the absence of YsxC, the r-protein uL2 binds to the 44.5S_YsxC_ particle; however, it does so unstably and wobbles at its binding site. In the presence of a non-hydrolyzable GTP analogue (GMPPNP), YsxC displaces uL2 and binds to uL2 binding site. The binding affinity of YsxC for the 44.5S_YsxC_ particle is higher than that for the 45S_RbgA_ and 45S_YphC_ assembly intermediates, and the prebinding of YsxC to the 44.5S_YsxC_ particle improves the subsequent binding of RbgA and YphC. This supports a model in which YsxC is the first factor to bind to the 44.5S_YsxC_ particle, and it is followed by the binding of RbgA and YphC to promote further maturation of the 44.5S_YsxC_ particle toward the mature 50S subunit. Using cryo-electron microscopy (cryo-EM), we describe how YsxC binds to the 44.5S_YsxC_ particle and controls the timing of rRNA helices folding, forming a “primordial” binding site that includes several of the rRNA helices critical for forming the uL2 binding site. Once YsxC is released, uL2 binds to this “primordial” binding site, and the remaining helices that stabilize uL2 fold, and the entire region adopts the mature conformation. Overall, these results suggest that YsxC serves as a placeholder factor for r-protein uL2 and provide the first example of the involvement of such a factor in the ribosome assembly process in bacteria.

## Materials and methods

### Bacterial strains

The 44.5S_YsxC_, 45S_YphC_, and 45S_RbgA_ particles and 50S subunits were purified from the YsxC-depleted (RB260), YphC-depleted (RB290), RbgA-depleted (RB301), and IF2-depleted (RB419) *B. subtilis* strains, respectively. The generation of these strains has been previously described [21, 27]. In these strains, the *ysxC, engA* (which encodes for YphC), *ylqF* (which encodes for RbgA), and *infB* (which encodes for IF2) genes are placed under the control of isopropyl-β-d-thiogalactopyranoside (IPTG) inducible P_spank_ promoter. In the absence of IPTG, these cells accumulate large amounts of the various assembly intermediates or 50S subunits (RB419).

### Purification of ribosomal particles

To purify the 44.5S_YsxC_ particles used in cryo-EM and microscale thermophoresis (MST), the YsxC-depleted (RB260) *B. subtilis* strain [21] was first grown at 37°C on LB agar plates supplemented with 5 µg/ml chloramphenicol and 1 mM IPTG. To initiate the depletion, colonies from the plate were resuspended in 100 ml of pre-warmed LB medium containing 5 µg/ml chloramphenicol (initial A_600_ around 0.01–0.02) and grown to A_600_ 0.4–0.5 at 37°C. The culture was then used to inoculate 600 ml of fresh pre-warmed media (also supplememted with 5 µg/ml chloramphenicol) and incubated at 37°C with agitation until it reached a doubling time of 120 min, which usually corresponds to A_600_ 0.3–0.4. This protocol minimizes the appearance of genetic suppressors. Doubling times of cultures were calculated as DT = (*t*_2_ − *t*_1_) × [ln 2/(ln OD_600_@t2/ln OD_600_@t1)].

Cells were harvested by centrifugation at 4500 x *g* for 20 min and washed with 30 ml PBS buffer. The cell pellet was then resuspended in 15 ml of buffer A [20 mM Tris–HCl (pH 7.5), 60 mM NH4Cl, 10 mM Mg acetate, 0.5 mM ethylenediaminetetraacetic acid (EDTA), 3 mM β-mercaptoethanol, supplemented with cOmplete™, Mini, EDTA-free Protease Inhibitor Cocktail (Roche), and 20 μl RNase-free DNase (Roche)]. Cells were lysed by passing them four times through a cold French press set at 20 000 lbs/in², and cell lysate was clarified by centrifugation at 32 000 x *g* for 40 min. The clarified lysate was layered over a 1.1 M sucrose cushion in buffer A of equal volume and centrifuged at 110 000 × *g* for 16 h. The ribosome pellet was resuspended in 5–10 ml of buffer E [20 mM Tris–HCl (pH 7.5), 60 mM NH_4_Cl, 10 mM MgCl_2_, 0.5 mM EDTA, 7 mM β-mercaptoethanol] under gentle agitation before further centrifugation at 110 000 × *g* for another 16 h. Finally, the ribosomal pellet was resuspended in 0.5–1 ml of buffer E [10 mM Tris–HCl (pH 7.5), 15 mM Mg acetate, 60 mM NH_4_Cl, 3 mM β-mercaptoethanol]. Typically, 50–60 A_260_ units of crude ribosomes in a volume of 0.5–0.7 ml were loaded on top of a 35 ml 18%–43% sucrose gradient equilibrated in buffer E. The gradients were centrifuged at 60 000 × *g* for 16 h. Gradients were fractionated by monitoring the A_280_, and peaks corresponding to 44.5S_YsxC_ particles (Supplementary Fig. S1) were collected, pooled, and pelleted by centrifugation at 110 000 × *g* for 16 h. The pellets were resuspended in 100–150 μl of buffer E, flash-frozen with liquid nitrogen, and stored at −80°C for later use in cryo-EM experiments.

The 45S_RbgA_, 45S_YphC_ particles, and 50S subunits were purified as previously described [26–28].

### Protein overexpression and purification

The pET21b-*ylqF* plasmid, which is used to overexpress RbgA with a C-terminal His_6_-tag, and the pET15b-*yphC* plasmid, which is used to overexpress YphC with a cleavable N-terminal His_6_-tag, were generated as previously described [23, 27]. The overexpression and purification of RbgA and YphC have also been described [26].

The construction of the pET15b-*ysxC* plasmid for the overexpression of YsxC with an N-terminal His_6_ tag cleavable by thrombin protease has also been described [23]. To overexpress YsxC, *E. coli* BL21 (DE3) cells were transformed with pET15b-*ysxC*. Bacteria were grown in LB media at 37°C with 215 RPM agitation and supplemented with 100 µg/ml ampicillin. Protein overexpression was induced at an OD_600_ of 0.5 using 1 mM IPTG. After overnight incubation at 18°C, the cells were harvested by centrifugation at 4500 × *g* for 15 min, washed with PBS 1× buffer, and stored at −80°C for further use. For purification, cells were resuspended in lysis buffer (50 mM Tris–HCl at pH 7.5, 500 mM NaCl) supplemented with cOmplete™, Mini, EDTA-free Protease Inhibitor Cocktail (Roche). We used four consecutive passes through a cold French press at 20 000 psi to induce cell lysis. Cell debris was pelleted by centrifugation at 32 000 × *g* for 45 min, and the supernatant was further cleaned by filtering through 0.45 µm and 0.22 µm syringe filters. The resulting clarified and filtered lysate was manually loaded onto a 5 ml HisTrap™ column (Cytiva) pre-equilibrated with binding buffer (50 mM Tris–HCl at pH 7.5, 500 mM NaCl, 20 mM imidazole) at a flow rate of 0.5 ml/min. The column was washed with binding buffer, and YsxC was eluted using a gradient of 20 to 600 mM imidazole. Fractions containing YsxC were pooled and dialyzed overnight against ion-binding buffer (50 mM Tris– HCl at pH 7.5, 100 mM NaCl, 2.5mM CaCl2, 5% glycerol), supplemented with 25 U/ml of thrombin protease to cleave the His_6_ tag. Any precipitated protein was removed by centrifugation, and the dialyzed protein was loaded onto a HiTrap™ SP HP (Cytiva) cation exchange column equilibrated with 50 mM Tris–HCl at pH 7.5, 100 mM NaCl, and 5% glycerol. Subsequently, YsxC was eluted using a gradient from 100 mM to 1 M NaCl gradient.

Fractions containing the protein were verified by sodium dodecyl sulfate–polyacrylamide gel electrophoresis (SDS–PAGE), pooled, concentrated, and exchanged into the storage buffer [50 mM Tris–HCl (pH 7.5), 400 mM KCl, 10 mM MgCl_2_, 2 mM DTT, 5% glycerol] using a 10 kDa cut-off centrifuged concentrator (Amicon). Protein purity was assessed by SDS–PAGE. Lastly, the concentrated YsxC was supplemented with cOmplete™, Mini, EDTA-free Protease Inhibitor Cocktail (Roche) and glycerol to reach 20%, quantified, frozen in liquid nitrogen, and stored at −80°C. YsxC was used in this buffer for the cryo-EM experiments.

For MST experiments, cells overexpressing YsxC were resuspended in 20 mM Na_2_HPO_4_ (pH 7.5), 500 mM NaCl, and 20 mM imidazole supplemented with cOmplete™, Mini, EDTA-free Protease Inhibitor Cocktail (Roche), and lysis was done by four consecutive passes through a cold French press at 20 000 psi. Cell debris was pelleted by centrifugation at 32 000 × *g* for 45 min, and the supernatant was further cleaned by filtering through 0.45 µm and 0.22 µm syringe filters. The clarified lysate was manually loaded onto a 1 ml HisTrap™ column (Cytiva) pre-equilibrated with 20 mM Na_2_HPO_4_ (pH 7.5), 500 mM NaCl, and 20 mM imidazole. The column was washed with the same buffer, and proteins were eluted using a 20–500 mM imidazole gradient. Fractions containing the protein were pooled and dialyzed overnight against 20 mM Na_2_HPO_4_ (pH 7.5), 100 mM NaCl buffer supplemented with 25 U/ml of thrombin protease. Dialyzed proteins were centrifuged at 12 000 × *g* for 10 min and subsequently loaded onto a HiTrap™ SP HP (Cytiva) cation exchange column and eluted using a 100 mM to 1 M NaCl gradient. Protein purity was assessed by SDS–PAGE, and the proteins were then labeled for MST experiments according to the manufacturer’s instructions. Labeled proteins were buffer exchanged to buffer E supplemented with 250 mM NaCl, quantified, the degree of labeling was calculated, and were then aliquoted, flash frozen in liquid nitrogen, and stored at −80°C for further use.

### MST experiments

YsxC, RbgA, and YphC were labeled using the second-generation Red-NHS dye (NanoTemper Technologies). All labeling reactions and MST measurements followed the protocols previously described for RbgA and YphC [26] and were applied in the same manner to YsxC. For measuring the binding affinity of YsxC, RbgA, and YphC, we used a labeled concentration of 60 nM and a concentration range for the assembly intermediates (44.5S_YsxC_, 45S_RbgA_, or 45S_YphC_ particles) from 2 µM to 61 pM, 4 µM to 122 pM, or 8 µM to 244 pM, depending on the experiment and as indicated in graphs in Fig. 2 and Supplementary Figs S5 and S6. In pre-binding experiments, a mix containing the same concentration (2 or 4 µM, depending on the experiment) of unlabeled factor and 44.5S_YsxC_ particles was prepared in MST buffer [10 mM Tris–HCl, (pH 7.5), 60 mM NH_4_Cl, 15 mM MgCl_2_, 1 mM DTT, 0.05% Tween-20] supplemented with 1 mM GMPPNP and incubated for 1 h before performing serial dilution. When YsxC was part of the reaction, either as the labeled protein or as a pre-binder to the 44.5S_YsxC_ particles, the MST buffer also contained 100 mM KCl in addition to the other components. The labeled protein was then added to each tube to achieve a final concentration of 60 nM and subsequently incubated for 1 h before being loaded into the glass capillaries. Measurements were conducted using a Monolith NT.115 instrument (NanoTemper Technologies) set at 25°C with an LED power of 20% and medium MST power. The dissociation constants were determined by plotting the difference in normalized fluorescence (Δ*F*_norm_ [‰] = *F*_1_/*F*_0_) against the logarithm of the various concentrations of 45S assembly intermediates. *F*_1_ and *F*_0_ represent the hot and cold regions of the thermophoresis traces. The resulting *K*_d_ values were calculated from three independently conducted experiments using the NanoTemper MO.Affinity analysis software (version 2.3).

### Cryo-electron microscopy

To prepare the grids containing only 44.5S_YsxC_ particles, we prepared a dilution of these particles at a concentration of 180 nM in buffer E. Depending on the experiment, the particle dilution was either directly applied to the electron microscopy grid or first incubated at 37°C for 15 min before being applied to the grid.

To visualize the YsxC-GMPPNP and YsxC-GTP-treated 44.5S_YsxC_ particles, we prepared a reaction mixture containing 1.8 µM of 44.5S_YsxC_ particles and 18 µM of YsxC, and incubated it at 37°C for 15 min. For the YsxC-GMPPNP-treated reaction, the reaction buffer included 10 mM Tris–HCl (pH 7.5), 15 mM MgCl_2_, 50 mM NH_4_Cl, and 2 mM GMPPNP (buffer Bcryo). In the case of the YsxC-GTP-treated 44.5S_YsxC_ particles, the reaction was performed in buffer E supplemented with 2 mM GTP. Once the incubation was completed, the reaction mixtures were diluted 10-fold in either buffer Bcryo (YsxC-GMPPNP-treated sample) or buffer E (YsxC-GTP-treated sample), which were supplemented with 2 mM of the nucleotide and 1.8 µM of YsxC.

Cryo-EM grids (c-flat CF-2/1–3Cu-T) used for all samples were prepared by evaporating a continuous layer of carbon (5–10 nm) to minimize the exposure of the ribosomal particles to the air-water interface. Just before applying the samples, the grids were washed in chloroform for 2 h and treated with glow discharge in air at 5 mA for 20 s. In all instances, a volume of 3.6 µl of sample was applied to the grid before they were vitrified in liquid ethane using a Vitrobot Mark IV (Thermo Fisher Scientific Inc.), with a blotting time of 3 s and a blot force of +1. The Vitrobot chamber was set to 25°C and maintained at 100% relative humidity. All datasets were collected at FEMR-McGill using a Titan Krios microscope at 300 kV, equipped with a Gatan BioQuantum LS K3 direct electron detector. The software used for data collection was SerialEM [29]. Images were captured in counting mode according to the parameters detailed in Supplementary Tables S1–S4.

### Image processing

All image processing steps were performed using CryoSPARC v4. Cryo-EM movies underwent beam-induced motion correction with Patch Motion Correction, applying default settings that included using information up to 5 Å resolution for frame alignment, a B-factor of 500, and a calibrated smoothing constant of 0.5 applied to the trajectories. All frames in the movies contributed to the production of the merged micrograph. CTF parameter estimation was carried out using Patch CTF estimation in the program. The minimum and maximum resolutions considered for estimating the CTF parameters were 25 and 4 Å, respectively, while the minimum and maximum defocus values were established at 1000 and 50 000 Å. Images with an estimated resolution of 6 Å or better were retained for further processing. The curated micrographs were divided into exposure groups using exposure group utilities.

In all particle datasets, particle picking on the selected micrographs was performed in two steps: 500 randomly selected micrographs were initially picked using Blob Picker with a circular blob and a minimum and maximum particle diameter of 256 and 290 Å, respectively. The selected particles were used to generate templates for subsequent template picking across all micrographs. The maximum resolution considered for particle selection in the micrographs was 20 Å. The angular sampling employed was 5 degrees, and the distance between particles was set to 1 (in units of particle diameters). Picked particles were extracted using a box size of 420 pixels. Particle curation was then conducted using 2D classification (2 cycles) and ab initio reconstruction routines. In the 2D classification step, we requested 50 classes, selecting 0.85 and 0.99 as the inner and outer window radii. The maximum resolution considered in the images was 6 Å, and we used 2 for the initial uncertainty factor. The circular mask diameter was 382 Å, while the remaining settings were maintained at their default parameters. The selected 2D classes were further curated through a second round of 2D classification using the same parameters. Before the curated particles underwent ab initio reconstruction, their motion correction was enhanced using local motion correction. For the ab initio reconstruction, we selected 0.85 and 0.99 as the inner and outer window radii, requesting 2 to 3 classes and considering a maximum and minimum resolution of 35 and 12 Å. The number of iterations before and after annealing started and ended was set to 200 and 300, respectively, and the increase of Fourier radius at each iteration was 0.04. All other parameters for this routine were maintained at their default settings and values.

The curated set of particles was used to generate a consensus refinement cryo-EM map using Non-Uniform Refinement under default settings with C1 symmetry, including optimized per-particle defocus and optimized per-exposure group CTF parameters. This 3D reconstruction was used to estimate the maximum resolution attainable with the data and to obtain a consensus cryo-EM map that was subsequently used to generate a mask for the 3D classifications.

Particle heterogeneity was examined in all datasets through 3D classification analysis using full-size particles. The 3D classification analysis routines were consistently executed with “Simple” as the initialization mode (initial models are derived from randomly selected particle subsets), requesting 3 classes with a maximum resolution of 8 or 10 Å, depending on the dataset. The number of O-EM epochs for online expectation optimization was set to 5, while all other settings utilized default values. The number of iterative hierarchical 3D classifications was typically two and is detailed for each dataset in Supplementary Figs S2, S7, S10, and S13. In all classifications, a solvent mask created with volume tools was employed, either from the consensus map in the first iteration of classifications or from a previously obtained class for subsequent classification iterations. The resulting maps from these 3D classifications were visually inspected in Chimera [30, 31], and clusters of particles representing similar assembly intermediates were merged.

In cases where we needed to investigate the presence or absence of uL2 or YsxC bound to the 44.5S_YsxC_ particles and analyze the multiple binding positions of uL2, we employed focused classification using a spherical focus mask around the uL2/YsxC binding site. The mask for these classifications was initially created using UCSF Chimera to segment the density in the selected region. Afterward, we utilized volume tools within CryoSPARC to dilate and soft-pad the mask by 6 pixels before generating it. The focused classification was then conducted through 3D classification analysis using the full-size particles, considering a maximum resolution of 4 Å. During the focused 3D classification step, the initialization mode was switched to “Input” to utilize the previous homogeneous reconstruction as the initial volume for all requested classes in the new iteration. The number of O-EM epochs performed during online expectation optimization was set to 5, with default values applied to all other settings. The placement of the focus classification stage within the overall classification strategy is detailed in Supplementary Figs S2, S7, S10, and S13.

Homogeneous groups of particles were used to produce high-resolution cryo-EM maps. To this end, particles from each class were first subjected to ab initio reconstruction, requesting one class and using the same parameters described above. The resulting map was then used as the initial model for a Non-Uniform Refinement run under default settings with C1 symmetry, incorporating options for optimized per-particle defocus, optimized per-group CTF parameters, Fit Spherical Aberration, Fit Tetrafoil, and Fit Anisotropic Magnification activated. Average resolution estimation and local resolution analysis were done using cryoSPARC with the gold-standard approach [32]. Cryo-EM map visualization was conducted in UCSF Chimera and Chimera X [30, 31].

### Molecular model building

All atomic models were manually built using WinCoot v 0.9.8.1 [33] following this procedure. The *B. subtilis* mature 50S subunit molecular model (PDB 8QPP) [34] served as the initial model and was fitted through rigid-body docking in UCSF Chimera [30]. Areas of the model not represented by the cryo-EM density were removed. This initial model was improved by rounds of real-space refinement in Phenix [35] and manual model building in Coot [33, 36]. The evaluation of the final models was done with Phenix cryo-EM Comprehensive validation tool and the Molprobity server [37, 38] (Supplementary Tables S2 and S3). The resulting molecular models were used for figure preparation with UCSF Chimera [30], Chimera X [31, 39], and Photoshop (Adobe). To produce the molecular models of the 44.5S_YsxC_ particle bound to YsxC, we used the molecular model of YsxC obtained through X-ray crystallography (PDB 1SVI) [40]. The YsxC molecular model was initially fitted by rigid-body docking in UCSF Chimera and real-space refined in WinCoot [33].

## Results

### Depletion of YsxC causes the accumulation of immature 50S particles with uL2 unstably bound

**Figure 1.**
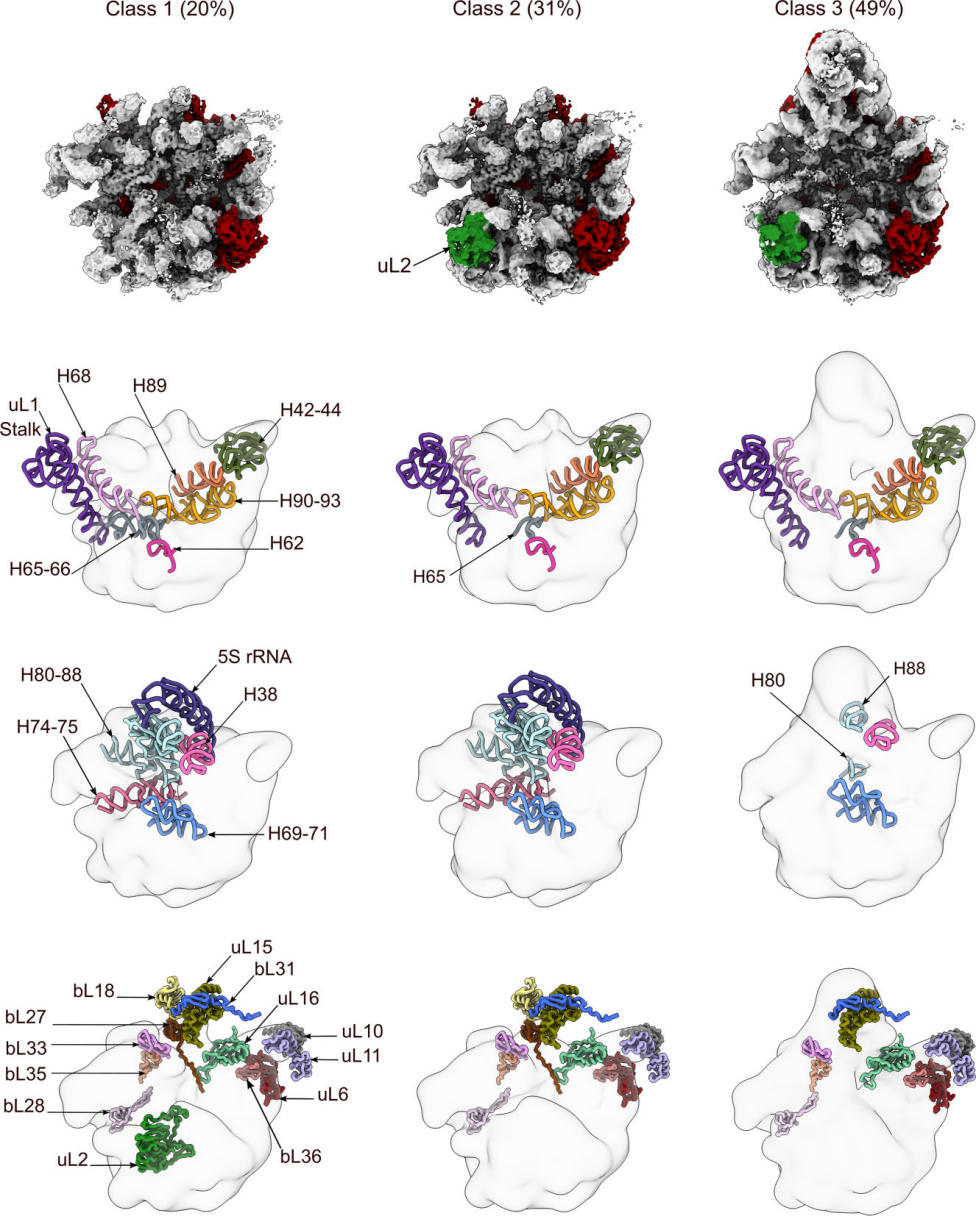
Cryo-EM structures of the 44.5S_YsxC_ particle. The top panels display the cryo-EM structures of the three classes of 44.5S_YsxC_ particles that accumulate in cells when YsxC is depleted. The percentage of particles for each class is indicated at the top. The rRNA helices and r-proteins are colored light gray and red, respectively. The r-protein uL2 is marked with an arrow and shown in forest green. The diagrams in the middle panels illustrate the rRNA helices for which corresponding density was not found across the three classes. The rRNA helices are represented in various colors, and their names are indicated by arrows. The bottom panel shows the r-proteins missing from each class and for which corresponding density is absent in the cryo-EM map.

To investigate the function of YsxC in the maturation of the 50S subunit, we purified the 44.5S_YsxC_ particles that accumulate in *B. subtilis* under YsxC depletion conditions [21, 23] (Supplementary Fig. S1) and analyzed them using cryo-EM. Single-particle and image classification approaches (Supplementary Fig. S2) applied to the particle images revealed that the 44.5S_YsxC_ particles are arrested in three distinct maturation states, which we name from class 1 to class 3 (Fig. 1, top panel).

We calculated a cryo-EM map for each state (Fig. 1, top panels), with resolutions ranging from 2.7 to 2.9 Å (Supplementary Fig. S3). A structural characteristic common to all classes was the immature state of their functional core, comprised of the A (H38, H89, and H90–92), P (H69, H71, H93), and E (H68) sites and the uL1 stalk (H76–H78). The densities corresponding to helices H62 and H65 around the uL2 binding site were also absent in all three classes. H66 in this same region was missing in class 1 but was visible and shifted in position compared to its mature location in classes 2 and 3 (Fig. 1, middle panels).

The main difference between the classes is that classes 2 and 3, which together capture 80% of the total particles, show an apparent density for uL2. However, uL2 is absent in class 1 (Fig. 1, top and bottom panels). Moreover, class 3, capturing 49% of the particles, features an apparent central protuberance (CP) formed by H80–H88 and 5S rRNA. In contrast, classes 1 and 2 do not exhibit CP or densities for helices H74–75 at the base of uL1. Finally, helices H42–44, which form the bL12 stalk, are not visible in any class (Fig. 1, middle panels).

In addition to the differences observed between classes concerning the presence or absence of uL2, we found that none of the classes show density for most of the r-proteins localized at the base of the CP, including uL16, bL28, bL33, bL35, and bL36. This indicates that this prominent structural domain is still being formed even in class 3. The r-proteins that are part of the CP, including uL15 and bL31, were also absent in all classes. Moreover, classes 1 and 2, which lack CP, also did not contain bL18 and bL27. Finally, uL10 and uL11, which cover the top of the b12 stalk, as well as uL6 at its base, were also not observed in any cryo-EM maps (Fig. 1, bottom panel).

Local resolution analysis of the cryo-EM maps revealed that the entirety of the body domain in the three classes of particles was solved to high resolution (Supplementary Fig. S3). However, the local resolution for uL2 and the region surrounding the r-protein uL2 in classes 2 and 3 was significantly lower, suggesting that uL2 is either moving or adopting a variable conformation. Further classification of the particles in these two classes, along with focused refinement in the uL2 area (Supplementary Fig. S2), revealed multiple subclasses, each demonstrating a different binding orientation for uL2 to the 44.5S_YsxC_ particles (Supplementary Fig. S4). We concluded that uL2 in the 44.5S_YsxC_ particles, which were not previously exposed to YsxC, wobbles and sits unstably at its binding sites.

### Binding behavior of YsxC to critical 50S assembly intermediates

**Figure 2.**
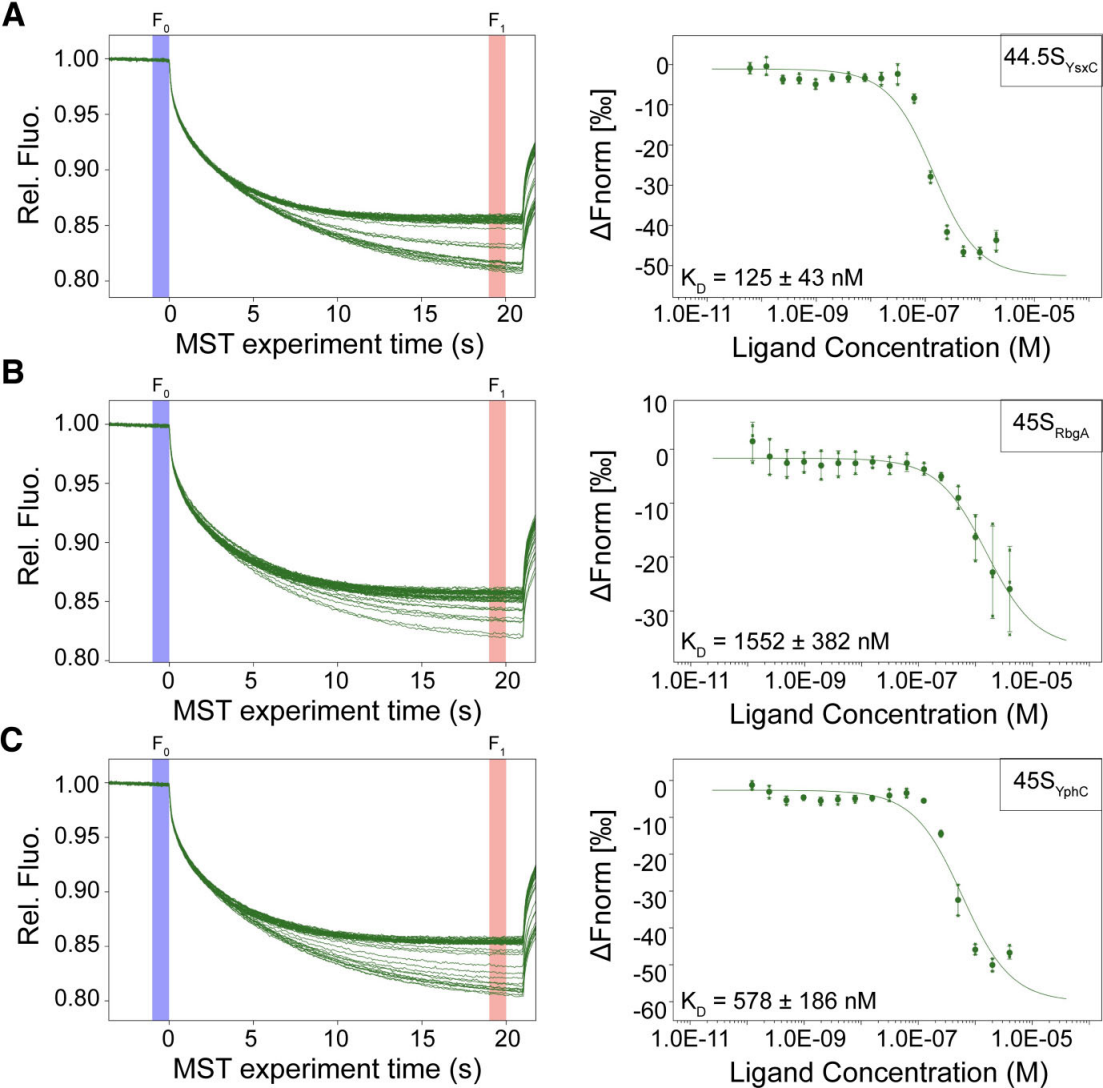
Binding affinity measurements of YsxC to the 44.5S_YsxC_ and 45S assembly intermediates. MST experiments were conducted to measure the binding affinity of YsxC to the 44.5S_YsxC_ (**A**), 45S_RbgA_ (**B**), and 45S_YphC_ (**C**) particles. All MST assays maintained a constant concentration of 60 nM for the labeled YsxC across all capillaries, while the assembly intermediates (44.5S_YsxC_, 45S_RbgA_, or 45S_YphC_ particles) varied in concentration from 2 µM to 61 pM. The plots on the left illustrate the thermophoretic mobility traces of the MST reactions, showing individual traces for each ribosomal particle concentration and highlighting the *F*_0_ (blue) and *F*_1_ (red) regions used to calculate binding. The YsxC binding plots (right panels) represent Δ*F*_norm_ (*F*_1_/*F*_0_) versus particle concentration. The *F*_norm_ curves were fitted using the law of mass action to derive *K*_d_ values. Dots indicate the average from the three replicates at each concentration, and error bars represent the standard deviation.

The accumulation of the 44.5S_YsxC_ particles in the cell under YsxC depletion conditions suggests that these particles represent the assembly intermediate in which YsxC binds to help advance the maturation process. Previous research has already established that YsxC can bind to the 44.5S_YsxC_ particles using filtration assays [23]. Here, we measured the binding affinity between YsxC and the 44.5S_YsxC_ particles using MST [41] in the presence of the non-hydrolyzable GTP analogue, GMPPNP. We monitored the thermophoretic mobility of fluorescently labeled YsxC at varying concentrations of the 44.5S_YsxC_ particles and determined that the *K*_d_ value of the assembly factor for the intermediate was 125 ± 43 nM (Fig. 2A).

It has been hypothesized that YsxC may assist in the assembly of the 50S subunit in conjunction with RbgA and YphC, two other assembly factors [4, 23]. During 50S assembly, RbgA and YphC bind to the 45S_RbgA_ and 45S_YphC_ particles. These two assembly intermediates are structurally similar [24, 26] and constitute a critical node where multiple parallel assembly pathways converge [4]. Our cryo-EM analysis (Fig. 1) shows that the 44.5S_YsxC_ particle represents an earlier assembly intermediate than the 45S particles, based on the fact that uL2 is not securely bound and it adopts a variable conformation. Therefore, we investigated whether the more mature 45S particles still preserve the ability to bind YsxC. Our MST experiments determined that the affinities of YsxC for the 45S_RbgA_ and 45S_YphC_ particles were ∼12 and 5 times worse, respectively, than the affinity exhibited for the 44.5S_YsxC_ particles (Fig. 2B and C). This result suggests that YsxC assists in the assembly of the 50S subunit by primarily binding to the 44.5S_YsxC_ particles, and most likely before this assembly intermediate evolves into the more mature 45S particle.

To establish whether RbgA or YphC may affect the binding of YsxC to the 44.5S_YsxC_ particle, we first determined if RbgA and YphC could bind to the 44.5S_YsxC_ particle and assessed their affinity for this particle. We found that YphC (Supplementary Fig. S5A) and RbgA (Supplementary Fig. S5B) were able to bind to the 44.5S_YsxC_ particle with similar affinity to their cognate assembly intermediates, the 45S_RbgA_ and 45S_YphC_ particles [24, 26]. Significantly, the prebinding of either YphC or RbgA to the 44.5S_YsxC_ particle did not dramatically change the binding affinity of YsxC to the 44.5S_YsxC_ particle (Supplementary Fig. S5C). However, the prebinding of YsxC to the 44.5S_YsxC_ particle significantly improved the subsequent binding of YphC (Supplementary Fig. S6A) and RbgA (Supplementary Fig. S6B). We also found that the previously described binding synergy between RbgA and YphC, observed when these factors bind to the 45S_RbgA_ and 45S_YphC_ particles [26], was also observed in the 44.5S_YsxC_ particle. Prebinding RbgA to the 44.5S_YsxC_ particle increased YphC’s binding affinity for this particle by ∼2-fold (Supplementary Fig. S5A). In the reverse reaction, prebinding of YphC improved RbgA’s binding affinity to the 44.5S_YsxC_ particle by ∼3-fold (Supplementary Fig. S5B).

Overall, these results, together with previously published data [26], align with a model in which YsxC assists in the assembly of the 50S subunit by primarily binding to the 44.5S_YsxC_ particles. YsxC is the first factor to bind, and this binding event favors the subsequent binding of RbgA and YphC to the 44.5S_YsxC_ particle.

### YsxC is a placeholder for the ribosomal protein uL2

**Figure 3.**
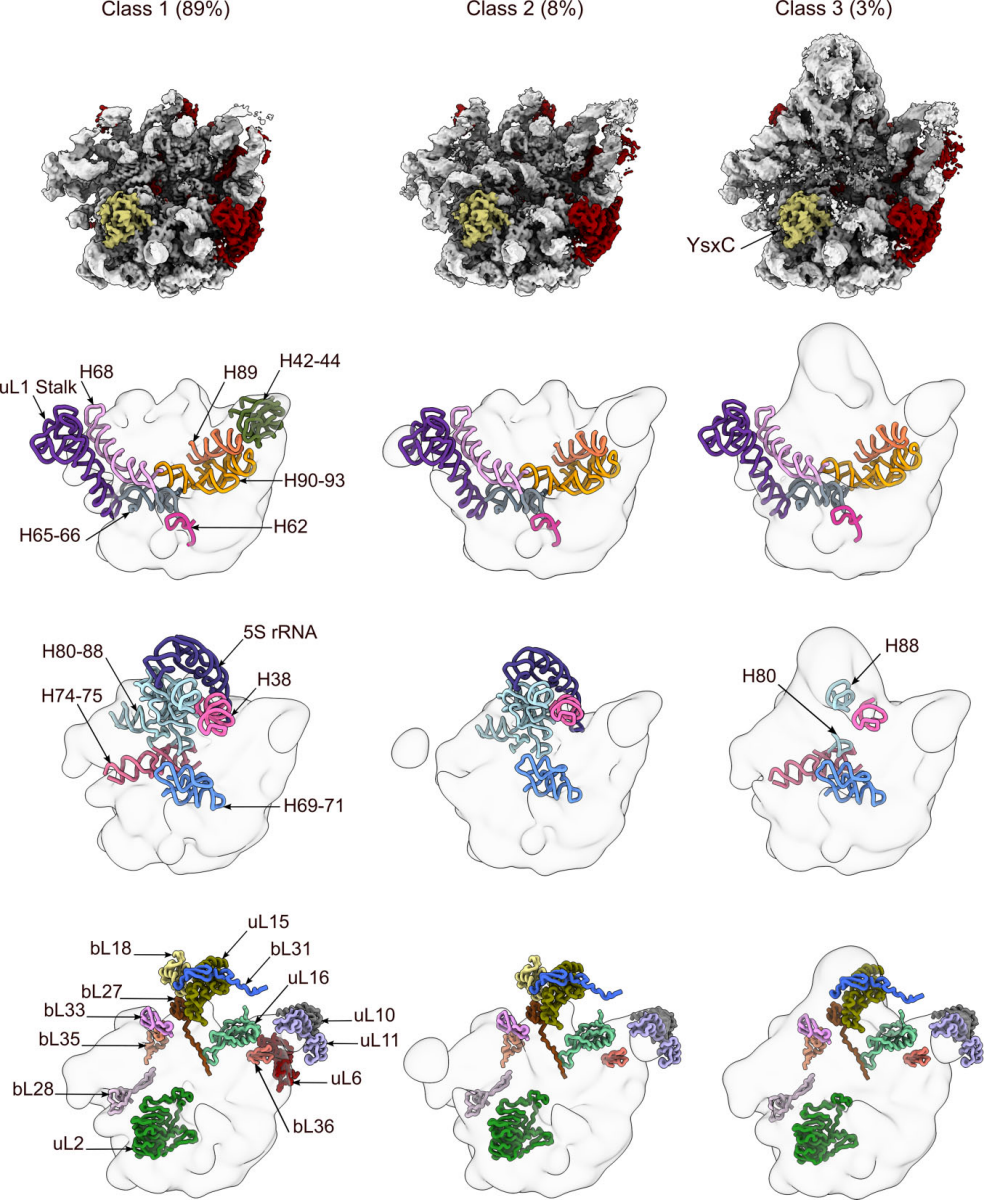
Cryo-EM structures of the 44.5S_YsxC_ particles in complex with YsxC. The top panel displays the cryo-EM maps of the three classes of 44.5S_YsxC_ particles observed after incubation with YsxC in a buffer containing GMPPNP. The percentage of particles for each class is indicated at the top. The rRNA helices and r-proteins are colored light gray and red, respectively. YsxC has replaced uL2 in the assembly intermediate, represented in khaki. The middle and bottom panels illustrate the rRNA helices and r-proteins absent from each class, following a layout similar to that of Fig. 1.

We used cryo-EM and single-particle analysis to identify the binding site of YsxC to the 44.5S_YsxC_ particle. In this experiment, we incubated the 44.5S_YsxC_ particles with YsxC in a buffer containing GMPPNP. Using image classification approaches (Supplementary Fig. S7), we identified three classes of particles, which we named from class 1 to class 3 (Fig. 3, top panel). The cryo-EM maps for these classes achieved resolutions ranging from 2.5 to 3 Å (Supplementary Fig. S8). Importantly, none of the three maps contained density for uL2. Instead, they showed an additional density bound in the uL2 binding site that resembled the crystal structure of YsxC [40]. Local resolution analysis of the three maps indicated that the density assigned to YsxC refined to similar high-resolution levels as the rest of the subunit body, suggesting that YsxC stably binds to the 44.5S_YsxC_ particle in a single conformation (Supplementary Fig. S8).

Similar to the classes observed for the 44.5S_YsxC_ particles alone (Fig. 1), these three classes also represented 50S assembly intermediates (Fig. 3, top panels). None of these classes show densities for the rRNA helices in the A, P, and E functional sites, as well as the uL1 stalk. Similarly, helices H62, H65, and H66, which are located near the uL2 binding site, were also not visible, and classes 1 and 2 lacked densities for the rRNA components forming the CP (Fig. 3, middle panels).

Class 1, representing the most immature particle, captured the majority of the particles (89%). This class was structurally similar to class 1 in the untreated 44.5S_YsxC_ particle sample (Fig. 1), except that YsxC was now bound in the previously vacant binding site for uL2 (Fig. 3, top panel). Class 2 captured only 8% of the particles and was structurally similar to class 1; however, in this class, helices at the base of uL1 (H74–75) and those forming the bL12 stalk (H42–44) had become visible (Fig. 3, middle panels). Finally, class 3 (Fig. 3, top panel) was structurally equivalent to class 3 in the untreated 44.5S_YsxC_ particle sample (Fig. 1), but in this class, uL2 had been replaced by YsxC. Regarding r-proteins, in addition to uL2 being replaced by YsxC across the three classes, the same r-proteins that were absent in the untreated 44.5S_YsxC_ particle sample (Fig. 1, bottom panel) were also missing in these classes (Fig. 3, bottom panel).

Even though the three classes of 44.5S_YsxC_ particles coexisting after the addition of YsxC were structurally similar to those observed before the YsxC treatment (Fig. 1, top panel), the percentage distribution of these classes changed drastically (Fig. 3, top panel). In the sample with the untreated 44.5S_YsxC_ particles, the percentage of particles exhibiting CP (class 3) constituted 49% of the total. However, with the addition of YsxC, the percentage of particles with CP dropped dramatically to 3%.

Overall, these data support a model in which YsxC serves as a placeholder protein for uL2. In this capacity, YsxC binds to the uL2 binding site in those 44.5S_YsxC_ particles that still lack uL2 or displaces this r-protein in the particles where it is already bound, preventing its rebinding. Upon YsxC binding, those particles that had partially matured the CP experience a reversion in the folding of this large structural motif and revert to an earlier intermediate state (classes 1 or 2), suggesting YsxC also exerts a “proofreading” role in removing particles that have already matured the CP ahead of the uL2 binding region.

### The binding of YsxC to the 44.5S_YsxC_ particles prevents the folding of rRNA helix 66 and induces conformational changes in the assembly factor

**Figure 4.**
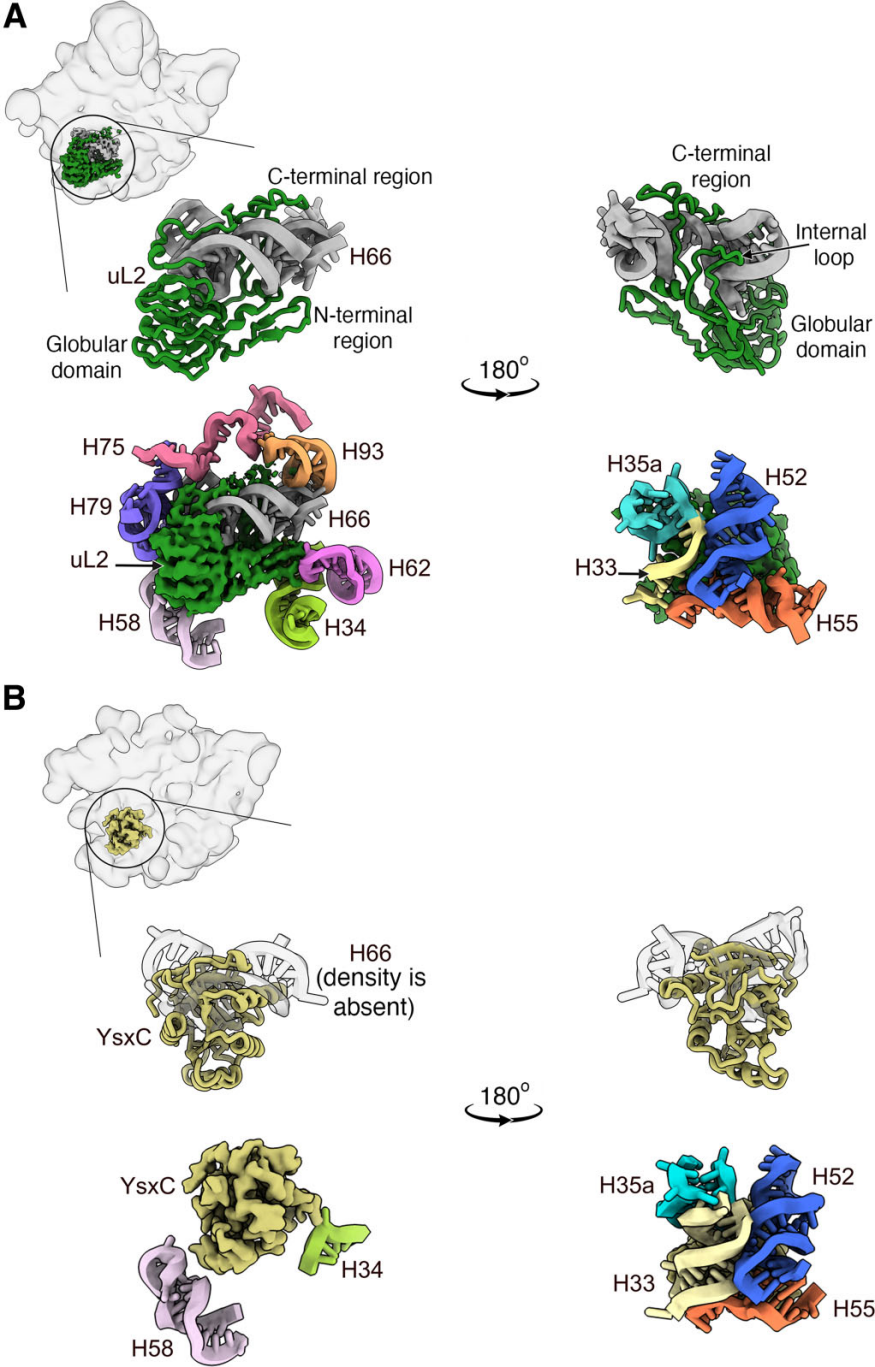
Binding context of uL2 and YsxC. (**A**) The top left panel shows the location of the r-protein uL2 (colored in forest green) in the 50S mature ribosomal subunit (PDB 8QPP). The zoomed-in views below show various views of uL2 to illustrate their interactions with the rRNA helices surrounding the binding site. The top two panels display a ribbon representation of the front and back views of uL2 and its interactions with the rRNA H66, which intertwines with various regions of uL2. The main regions of uL2 are labeled. The bottom panels present the cryo-EM density of uL2, segmented out, along with the rRNA helices interacting with uL2. The left panel represents the same front view of uL2, while the right panel shows the back view. (**B**) YsxC has a different folding than uL2, but it binds in the same location as the r-protein. The panel in the top left corner displays the 44.5S_YsxC_ particle class 1 with YsxC-GMPPNP bound (colored in khaki). The panels below present various views of YsxC and the rRNA helices interacting with it. The top panels depict a ribbon representation of YsxC alongside only the rRNA H66. This rRNA helix is shown as a transparent outline to indicate its conformation in the mature 50S subunit. This conformation is incompatible with YsxC being bound to the 44.5S_YsxC_ particle, and a density corresponding to H66 is absent in the cryo-EM map. The bottom panels illustrate the cryo-EM density of YsxC, segmented from the cryo-EM map, along with the rRNA helices interacting with YsxC. The left panel represents the same front view of YsxC, and the right panel shows the back view.

uL2 is a large r-protein that binds to the 50S subunit in the lower part of the body, beneath the uL1 stalk at the subunit interface (Fig. 4, top panel). The main portion of uL2 is a β-strand-rich extended globular domain that rests on the surface of the ribosomal subunit. However, this protein also includes the N-terminal region, an internal loop (Lys 31-Arg 63), and a C-terminal region, adopting extended conformations that insert into the 50S subunit and anchor the protein through interactions exclusively with the rRNA (Fig. 4A). In particular, the N-terminal region and globular part of uL2 interact with H34, H58, H62, and H79, while the internal loop and C-terminal unfolded regions wrap around H66 and also interact with H33, H35a, H52, H55, H75, and H93 (Fig. 4A).

In our study, we used the cryo-EM map of the YsxC-GMPPNP-treated 44.5S_YsxC_ particle class 1 (Fig. 3, top panel), which had the highest resolution, to explore the binding site of YsxC in the ribosomal particle at the molecular level. We discovered that YsxC inserts itself into the binding site of uL2 and partially occupies the location of H66, preventing this helix from adopting its mature conformation and becoming visible (Fig. 4B). Consequently, simultaneous binding of YsxC and uL2 is not possible. All the interactions facilitating the binding of YsxC with the ribosomal particle involve RNA helices (Fig. 4B). To establish these interactions, YsxC uses its surface with the highest density of exposed positively charged residues. The opposite side of YsxC, which is richer in negatively charged amino acids, is exposed to the solvent at the subunit interface (Supplementary Fig. S9).

In its binding site, YsxC contacts six of the rRNA helices also contacted by uL2, including H33, H34, H35a, H52, H55, and H58 (Fig. 4B). Importantly, the positions of these helices are not significantly different from those adopted in the mature 50S subunit. This result suggests that the main task of YsxC as a placeholder protein is to prevent H66 from adopting its mature conformation before uL2 binds. YsxC also stabilizes the other six rRNA helices that the protein contacts in a close-to-mature conformation, preventing them from exploring other conformations before uL2 binds and while other regions of the rRNA continue to fold. All these events likely contribute to the correct folding of the rRNA helices surrounding uL2 and, in turn, to the more efficient binding of uL2 to the assembling intermediate once YsxC is released.

**Figure 5.**
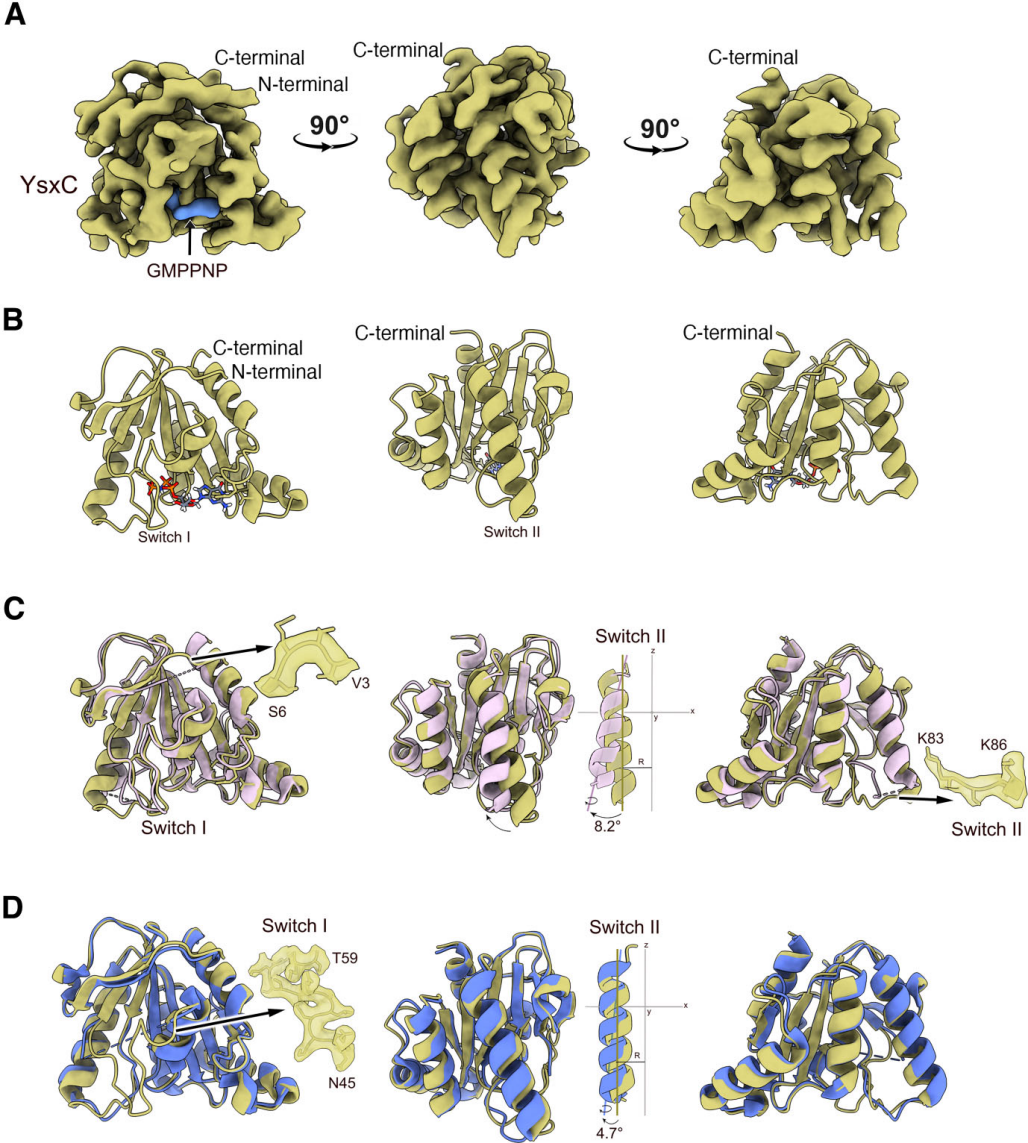
Structure of the YsxC-GMPPNP factor bound to the 44.5S_YsxC_ particle. (**A**) Three orthogonal views of the density corresponding to the YsxC-GMPPNP factor, after extraction from the cryo-EM map obtained for the YsxC-GMPPNP-treated 44.5S_YsxC_ particle (class 1). The protein’s N- and C-terminal ends, as well as the density corresponding to the GMPPNP nucleotide (colored in blue), are indicated. (**B**) Equivalent views to those in (**A**) of the YsxC-GMPPNP molecular model built from the cryo-EM structure. The N- and C-terminal ends, along with switch I and switch II of the protein, are indicated. We have also labeled the α-helices and β-strands forming the molecular model. (**C**) This panel shows the same views of the molecular model of YsxC-GMPPNP bound to the 44.5S_YsxC_ particle, derived from our cryo-EM structure, overlaid with the molecular model in light pink of the free YsxC-GMPPNP factor obtained through X-ray crystallography [40]. We also include an enlarged view of the density representing the N-terminal (left panel) and loop region of switch II (right panel) in YsxC, which are visible in the cryo-EM structure but not in the X-ray structure. The diagram in the middle panel highlights that α-helix 2, part of switch II, changes its orientation by 8.2° compared to the free YsxC-GTP state [40]. (**D**) Overlay of the structure of YsxC-GMPPNP bound to the 44.5S_YsxC_ particle obtained by cryo-EM with the free YsxC-GDP structure obtained by X-ray crystallography [40]. In the left panel, we include an enlarged view of the density representing switch I, which is visible in the cryo-EM structure but not in the X-ray structure. In the middle panel, we include a diagram to highlight that α-helix 2 changes its orientation, but only by 4.7° with respect to the free YsxC-GDP state [40].

The binding of YsxC to the 44.5S_YsxC_ particle exerts stabilizing effects on the assembly factor itself (Fig. 5A). YsxC shares a common fold with other small GTPases in the TRAFAC class. GTPases from this family fold into a central β-sheet flanked by α-helices (Fig. 5B). As the GTPase transitions between the GTP and GDP states, two distinct unfolded regions, known as switch I (residues 45–65) and switch II (residues 75–103), change conformation [40]. Our cryo-EM structure of YsxC bound to the 44.5S_YsxC_ particle (Fig. 3) mimics the GTP state of the enzyme since it was obtained in the presence of the non-hydrolyzable GTP variant, GMPPNP. In the X-ray structure of free YsxC with GMPPNP, the switch II (residue 83–86) and N-terminal (residue 3–6) regions of the protein are disorganized and not visible. However, these regions become stabilized and visible in the YsxC protein upon binding to the 44.5S_YsxC_ particle (Fig. 5C). Additionally, α-helix 2, which is part of switch II, also changes its orientation by 8.2° compared to the free YsxC GTP state [40] (Fig. 5C).

To study the conformational changes that YsxC undergoes upon GTP hydrolysis, we compared the structure of YsxC-GMPPNP bound to the 44.5S_YsxC_ particle with the structure of the free YsxC-GDP form obtained by X-ray crystallography [40]. Overlapping the molecular model of these two structures (Fig. 5D) revealed that the switch I region that is organized in the GMPPNP-bound form of YsxC becomes unstructured in the GDP-bound form. In addition, α-helix 2, forming part of switch II, changes its orientation but only by 4.7° [40].

Overall, these comparisons allowed us to conclude that upon binding the 44.5S_YsxC_ particle, the N-terminal, switch I, and switch II regions of YsxC become structured, and α-helix 2 in the switch II also moves. However, GTP hydrolysis mainly affects the conformation of switch I, which becomes unstructured. These conformational changes are likely part of the mechanisms for YsxC to regulate its binding and release from the 44.5S_YsxC_ particle.

### YsxC binding promotes the stable binding of uL2 to the 44.5S_YsxC_ particles and promotes further maturation of the assembly intermediate

**Figure 6.**
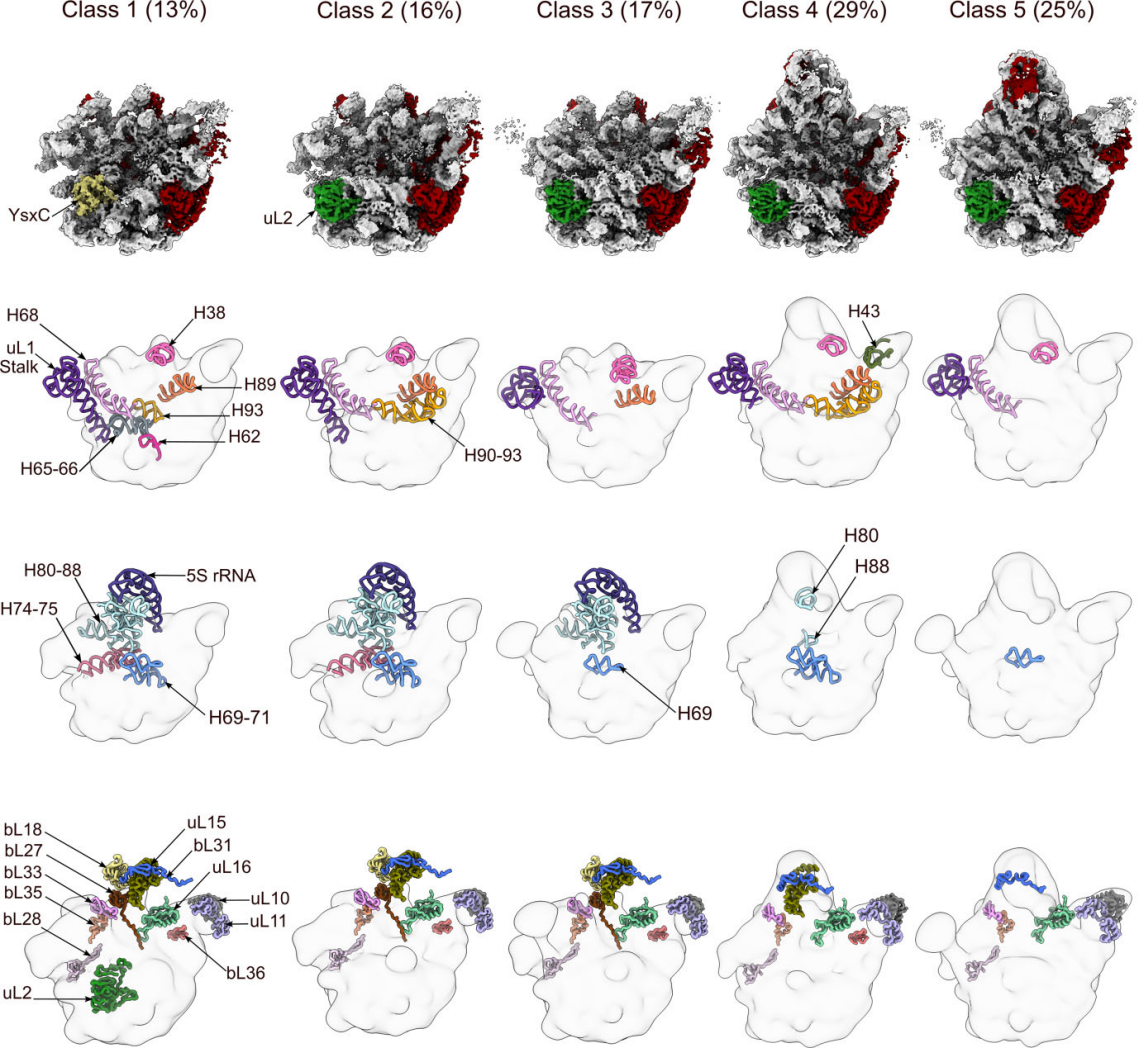
Cryo-EM structures of the GTP-YsxC-treated 44.5S_YsxC_ particles. The top panel shows the five classes of 44.5S_YsxC_ particles observed after incubation with YsxC and buffer containing GTP. The percentage of particles assigned to each class in the classification approaches is indicated at the top. The rRNA is shown in gray, and r-proteins are colored red. The YsxC protein bound in class 1 is colored khaki. The middle and bottom panels display the rRNA helices and r-proteins missing from each class, using a layout similar to Fig. 1.

Next, we tested the effect of YsxC treatment with GTP on the maturation of the 44.5S_YsxC_ particles. To achieve this, we incubated the 44.5S_YsxC_ particles at 37°C for 15 min in the presence of a 10-fold excess of YsxC in a buffer containing 2 mM GTP. Visualization and analysis of the maturation reaction using cryo-EM and image classification techniques (Supplementary Fig. S10) revealed that the 44.5S_YsxC_ particles evolved into five distinct classes after YsxC-GTP treatment (Fig. 6). Particles associated with each class produced a cryo-EM map that was refined to resolutions ranging from 2.8 to 3 Å (Supplementary Fig. S11).

Compared to the untreated 44.5S_YsxC_ particle dataset, we found that YsxC-GTP treatment increased the percentage of 44.5S_YsxC_ particles with uL2 bound (classes 2 to 5) from 80% to 87%. This small increase in the percentage of 44.5S_YsxC_ particles containing uL2 bound was expected, as additional uL2 was not added in the reaction along with YsxC and GTP. The remaining 13% of particles (class 1) still had YsxC bound. Importantly, the local resolution of the density representing uL2 in classes 2 to 5 was similar to other areas of the particle body (Supplementary Fig. S11). Analyzing the uL2 density from the four maps revealed that uL2 is bound to these classes in a single conformation (Supplementary Fig. S12), identical to that on the mature 50S subunit. Furthermore, YsxC treatment also triggered the progressive maturation of the 44.5S_YsxC_ particles (Fig. 6). Class 1 represents the more immature particles. YsxC is still bound to these particles, which also lack the CP (Fig. 6, top panel). This class is similar to class 1 in the YsxC-GPMPNP treated 44.5S_YsxC_ particle dataset (Fig. 3). Similarly, it still lacks densities for the rRNA helices in the CP (H80–88 and 5S rRNA), uL1 stalk, and helices H74–75 at the base of uL1. Consistent with our other cryo-EM maps with YsxC bound (Fig. 3), helices H62, H65, and H66 around the uL2 binding site are also not yet organized. Unlike class 1 in the YsxC-GPMPNP treated 44.5S_YsxC_ particle dataset (Fig. 3), particles in class 1 have already initiated the maturation of the A site with visible density for helices H90–92. The bL12 stalk is also present. However, the rRNA helices in the A (H89), P (H69, H71, H93), and E (H68) sites are still not visible (Fig. 6, middle panels). Class 2 is the first class in this dataset showing the effect of YsxC-GTP treatment with correctly accommodated uL2. This class is similar to class 2 in the untreated 44.5S_YsxC_ particles dataset (Fig. 1), but here, uL2 is stably bound, and the bL12 stalk is already matured (Fig. 6, middle panels). From class 2 to class 5, we observed the progressive maturation of the uL1 stalk base (H74-75), CP, A, and P sites. The CP is still absent in classes 2 and 3 but becomes progressively more complete in classes 4 and 5. The A and P sites are fully immature in class 2, but they progressively show more densities for the rRNA helices comprising these sites in classes 3 to 5. Only the E site remains immature in all classes (Fig. 6, middle panels), likely because it also requires the influence of other assembly intermediates, such as RbgA [24] and YphC [26]. An important consequence of the correct accommodation of uL2 in classes 2 to 5 is that H62, H65, and H66 around the uL2 binding site become properly organized, adopting their mature conformation (Fig. 6, middle panels).

Regarding r-proteins missing from 44.5S_YsxC_ particles in these classes (Fig. 6, bottom panel), we found that they did not differ from those also absent in the untreated (Fig. 1) and YsxC-GMPPNP-treated 44.5S_YsxC_ particle (Fig. 3) datasets. This result was anticipated since all these *in vitro* maturation reactions used purified 44.5S_YsxC_ particles, and no additional free r-proteins were introduced to the reaction. Nevertheless, uL6, located below the bL12 stalk, became stabilized in class 5, representing the most mature particles in the dataset (Fig. 6).

### YsxC increases the maturation efficiency for the last stages of assembly of the 50S ribosomal subunit

**Figure 7.**
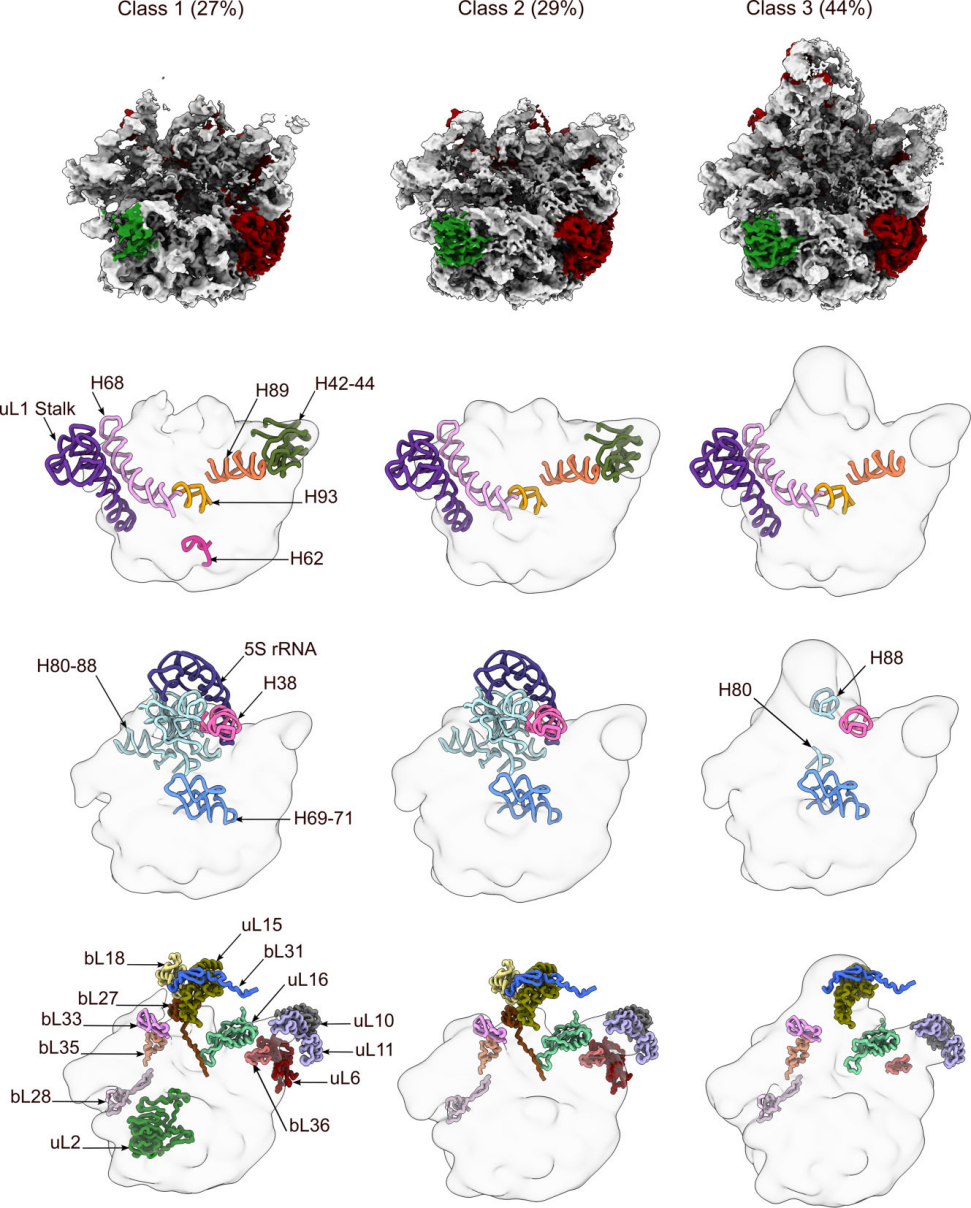
Cryo-EM structures of the 44.5S_YsxC_ particles after incubation in the absence of YsxC. The top panels display the three classes of 44.5S_YsxC_ particles observed after incubation at 37°C for 15 min, without adding YsxC to the buffer. The rRNA helices and r-proteins are colored light gray and red, respectively, while the r-protein uL2 is shown in forest green. The percentage of particles assigned to each class in the classification approaches is indicated at the top. The middle and bottom panels illustrate the rRNA helices and r-proteins absent from each class, using a layout similar to Fig. 1.

To determine whether YsxC is essential for the maturation steps observed in the 44.5S_YsxC_ particle, we incubated the 44.5S_YsxC_ particles at 37°C for 15 min without adding YsxC to the reaction. After incubation, we analyzed the ribosomal particles using cryo-EM and image classification methods (Supplementary Fig. S13) and found that the particles evolved into three classes. We generated cryo-EM maps for these three classes (Fig. 7, top panel) that refined to resolutions of 2.9 and 3.4 Å, respectively (Supplementary Fig. S14).

Structurally, classes 1 and 2 lacked CP and were similar to class 3 in the YsxC-GTP treated-44.5S_YsxC_ particles dataset (Fig. 6, top panel), while class 3 had CP and was similar to class 4 in the same dataset (Fig. 6, top panel). These particles were also missing the same r-proteins as their counterparts in the YsxC-GTP-treated 44.5S_YsxC_ particles dataset (Fig. 6, bottom panel). The density for uL2 was highly fragmented and nearly absent in class 1 (Fig. 7, top panel), but was present in classes 2 and 3. Helices H62, H65, and H66 surrounding uL2 were also found in these two classes (Fig. 7, middle panels). In addition, uL2 was partially accommodated, but bound in a similar manner to both classes (Supplementary Fig. S15). Additionally, the three classes exhibited partial maturation of the rRNA helices that form the A site (Fig. 7, middle panels). Nevertheless, the densities for these rRNA helices were fragmented (Fig. 7, top panel), indicating they had not reached a fully mature state.

These results suggest that the 44.5S_YsxC_ particles are not fully stalled in the assembly process, waiting for YsxC to bind and push them forward through maturation. The appropriate accommodation of uL2 and further maturation of the functional sites in the 44.5S_YsxC_ particles can occur spontaneously, but our results indicate that these maturation steps are more efficient in the presence of YsxC.

## Discussion

One of the results obtained in this study is the high-resolution cryo-EM structure of the 44.5S_YsxC_ assembly intermediate, which accumulates in the cells when the YsxC factor is depleted. The 45S_RbgA_ and 45S_YphC_ particles are two other assembly intermediates that accumulate upon depletion of RbgA and YphC, respectively [21, 27]. All three assembly intermediates share an identical protein complement [23, 28]. However, based on the obtained cryo-EM structure of the 44.5S_YsxC_ particle (Fig. 1) and the fact that uL2 is not securely bound, it appears that the 44.5S_YsxC_ particle represents an earlier assembly intermediate than the 45S_RbgA_ and 45S_YphC_ particles.

It has been suggested that the 45S_RbgA_ and 45S_YphC_ particles serve as a convergence point for all the parallel assembly pathways of the 50S subunit in *B. subtilis* [4]. The 45S_RbgA_ and 45S_YphC_ particles are in a “locked” state and require the binding of RbgA and YphC to continue their evolution to the mature state. The simultaneous binding of both factors to the assembly intermediate is favored, with the initial binding of RbgA promoting the subsequent binding of YphC [26]. Previous work [26] and the current experiments demonstrate that the binding affinity of RbgA and YphC for the 45S_RbgA_, 45S_YphC_, and 44.5S_YsxC_ particles is similar; thus, RbgA and YphC may bind as early as when the 44.5S_YsxC_ particle appears. Differently, YsxC shows a clear preference for the 44.5S_YsxC_ particle (Fig. 3), and the prebinding of YsxC to the 44.5S_YsxC_ particle significantly improved the binding of YphC (Supplementary Fig. S6A) and RbgA (Supplementary Fig. S6B). Therefore, the proposed model is that YsxC assists in the assembly of the 50S subunit by primarily binding to the 44.5S_YsxC_ particles. YsxC is the first factor to bind, and its binding favors the subsequent binding of RbgA and YphC to the 44.5S_YsxC_ particle that then evolves into the 45S particle and later into the mature 50S subunit after additional maturation steps.

These results also reveal that the role of YsxC is to ensure the proper folding of the binding region for uL2. We observed that YsxC occupies the binding site of uL2 and prevents H66, critical for uL2 binding, from adopting its mature conformation (Fig. 4B). In addition, the binding of YsxC stabilizes H33, H34, H35a, H52, H55, and H58 (Fig. 4B) in the position where they will eventually be contacted by uL2. The model emerging from this data suggests that YsxC acts as a placeholder to assist the maturation of the uL2 binding region by controlling the timing in the folding of rRNA helices H33, H34, H35a, H52, H55, and H58. By prioritizing the folding of these rRNA helices and delaying the formation of H66, YsxC prevents these helices from exploring other conformations that are not favorable for uL2 binding. Helices, H34 and H58, stabilized by YsxC at the subunit interface also create contact sites that guide uL2 incorporation. In this manner, YsxC creates a “primordial” binding site for uL2 that includes six out of the eleven rRNA helices forming the uL2 mature binding site (Fig. 4A). The obtained structures indicate that once YsxC is released, uL2 binds to this “primordial” binding site, and the remaining helices that stabilize uL2 (H62, H66, H75, H79, and H93) fold, and the entire region adopts the mature conformation (Fig. 6). Our results are consistent with recent time-resolved dimethyl sulfate footprinting, suggesting the uL2 binding region remains flexible until the late stages of the 50S subunit assembly [42], and previous cryo-EM experiments indicating that uL2 can join the assembly at different stages depending on the folding path [43]. Nevertheless, the role we describe here of YsxC functioning as a placeholder factor for r-protein uL2 constitutes the first example of such a factor’s involvement in the ribosome assembly process in bacteria.

An interesting observation in our study was that the binding of YsxC to the subpopulation of 44.5S_YsxC_ particles exhibiting a partially assembled CP caused this large structural landmark to disappear and the 44.5S_YsxC_ particle to revert to an earlier assembly intermediate. Notably, the observation of ribosome assembly intermediates reverting to an earlier maturation state is not unprecedented. An earlier study demonstrated that the mature 50S subunit reverts to a 45S-like particle upon removal of the uL16 r-protein [28]. This phenomenon of reversion in the maturation process is not limited to the 50S subunit. Multiple examples of maturation reversion have been described in the 30S subunit during assembly. One such study showed that treating *E. coli* 30S subunits with the Era assembly factor destabilized functionally essential regions of the 30S subunit, including key areas of the decoding center and platform domain [44]. A second study [45] has also shown that KsgA has a role in pruning 30S assembling subunits, in which helix 44 matured into an inactive conformation [46]. In this role, KsgA recognizes the inactive conformation of the 30S subunit, and upon binding, KsgA displaces helix 44 and a linker helix connecting the body domain of the particle with the head domain. The disruption of this helix uncouples the head, body, and platform domains, leading to partial subunit disassembly [45]. This observation suggested a “proofreading” role for KsgA during the assembly of the 30S subunit. In this role, KsgA provides “off-path” assembly intermediates that continue to mature in the absence of the factor and are not competent to reach the mature state, a second chance to reassemble into an active state, thereby increasing the overall fidelity of the assembly process. Similarly, the dramatic decrease of 44.5S_YsxC_ particles with central protuberance upon YsxC treatment suggests that YsxC also exerts a “proofreading” role in removing particles that have already matured the central protuberance ahead of the uL2 binding region. It is also consistent with the recent finding that rRNA H33 and H35a form long-range contacts with other regions of the ribosome [42].

YsxC, like RbgA and YphC, which also contribute to the maturation of the 50S subunit, is a GTPase. In the case of RbgA and YphC, the presence of GTP promotes binding to the 45S particles [21, 24, 26, 28, 47, 48]. However, GTP hydrolysis is not necessary for these two assembly factors to complete their function. Both factors facilitate the maturation of rRNA helices in the particle’s functional site upon binding, without the need for GTP hydrolysis. Hydrolysis of the nucleotide is essential to release the factor from the 45S particle. Conversely, regarding YsxC, the factor’s role is to control the timing in the folding of rRNA helices forming the binding site for uL2. Since the binding site for YsxC largely overlaps with the binding site of uL2, the final positioning of the r-protein in the mature conformation can only occur after the release of YsxC, which is triggered by GTP hydrolysis. Consequently, for YsxC, GTP hydrolysis is required for the factor to fulfill its role in the maturation of the 50S subunit.

This study provides new insights into the specific role that YsxC plays in the assembly of the 50S subunit. However, questions about YsxC still remain. An important observation is that continuous depletion of YsxC causes cell death, suggesting that this factor is essential [21]. Our experiment, in which we incubated the 44.5S_YsxC_ particles in the absence of YsxC, indicated that the assembly intermediate is not “locked” and some of the maturation steps facilitated by YsxC still happen spontaneously, although less efficiently. Therefore, our experiments do not explain the essentiality of this factor for cell survival. One possibility is that YsxC plays a critical role in ensuring the structural fidelity, proper timing, and coordination of late-stage ribosome assembly events. In its absence, misassembled or unstable intermediates may accumulate, ultimately leading to the production of non-functional ribosomes that are incapable of sustaining translation. Alternatively, the essential nature of YsxC may not be limited to its structural role in ribosome biogenesis. Like other GTPases involved in ribosome assembly, YsxC could participate in quality control mechanisms, act as a checkpoint factor that links ribosome maturation to the cell cycle, or engage in regulatory interactions beyond the ribosome that are indispensable for cell viability. These additional functions may not be recapitulated in our *in vitro* system, which isolates ribosome assembly from the broader cellular context. Further investigations will be required to determine whether the essentiality of YsxC stems solely from its contribution to ribosome assembly or also involves extraribosomal roles critical for bacterial growth and survival.

## Supplementary Material

gkaf1071_Supplemental_File

## Data Availability

The cryo-EM maps obtained in this study and the derived molecular models have been deposited in the Electron Microscopy Data Bank (EMDB) and the Protein Data Bank (PDB) with accession codes detailed in Supplementary Tables S1–S4.
