# Supplementary material for: YsxC is a placeholder for ribosomal protein uL2 during 50S ribosomal subunit assembly
Source: Nucleic Acids Res. 2025 Oct 28;53(20):gkaf1071. doi: 10.1093/nar/gkaf1071 (PMC12560758; doi:10.1093/nar/gkaf1071)
Supplement: gkaf1071_Supplemental_File [file gkaf1071_supplemental_file.pdf]

YsxC is a placeholder for ribosomal protein uL2 during 50S ribosomal subunit assembly.

Amal Seffouh[1,2], Dominic Arpin[1,2], Kaustuv Basu[1,2] and Joaquin Ortega[1,2*]

[1]Department of Anatomy and Cell Biology, McGill University, 3640 Rue University, Montreal, Quebec H3A 0C7, Canada, and the [2]Centre de Recherche en Biologie Structurale, McGill University, 3649 Promenade Sir William Osler, Montreal, Quebec H3G 0B1, Canada.

*Correspondence:

Joaquin Ortega, Department of Anatomy and Cell Biology, McGill University, Montreal, QC H3A 0C7 Canada. Phone: 1 (514) 398-5230. Email: joaquin.ortega@mcgill.ca

This supplement contains:

Supplementary Figures S1 to S15

Supplementary Tables S1 to S4

SUPPLEMENTARY FIGURES

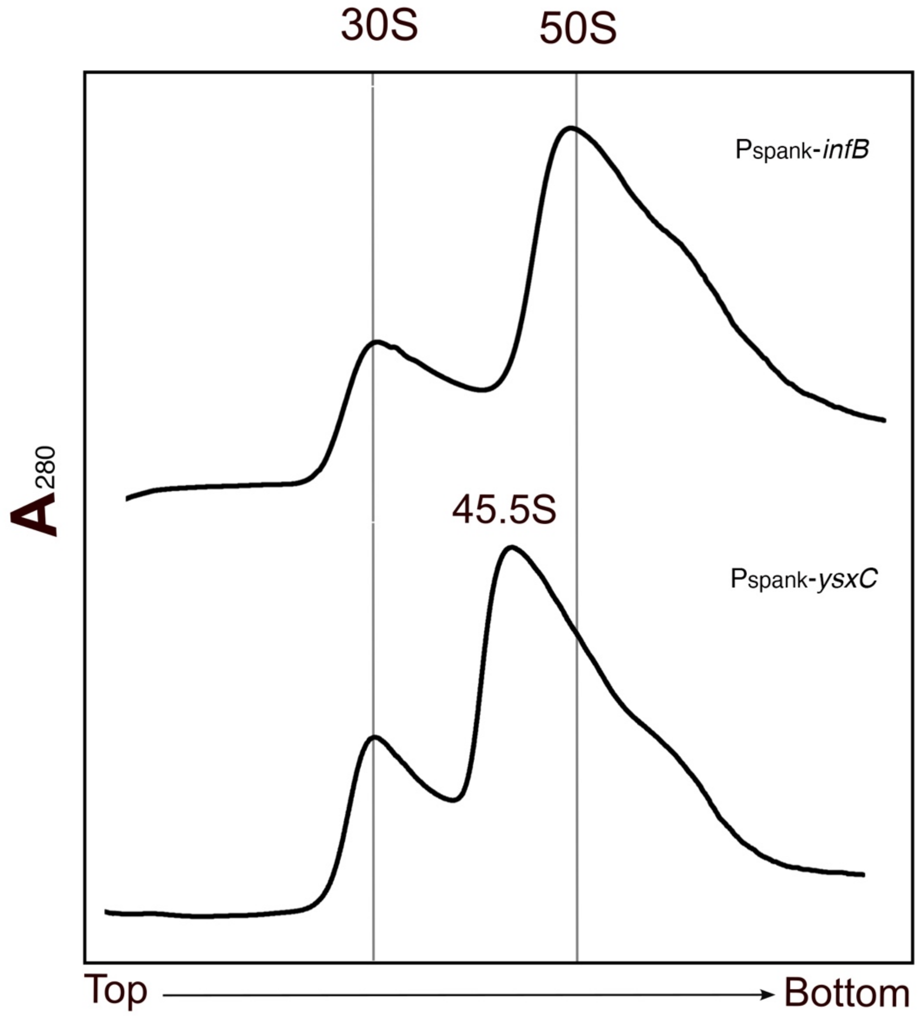

Supplementary Figure S1. ***Purification of the 44.5S$_{YsxC}$ particles.*** *The 44.5S$_{YsxC}$ immature ribosomal particles were purified from RB260 strain cells where the only copy of the ysxC gene was under the control of the IPTG-inducible promoter P$_{spank}$. Cells were grown in the absence of IPTG to generate YsxC depletion and induce the accumulation of the 44.5S$_{YsxC}$ particles. The assembly intermediates were purified by ultracentrifugation by using a 18-43% sucrose gradient. The bottom panel shows the profile of the sucrose gradient obtained by monitoring the absorbance at 280 nm through the gradient from the top to the bottom. Vertical lines indicate where the mature 30S and 50S particles migrate in the gradient, and they were calibrated by running an identical gradient (top panel) containing mature 50S and 30S subunits purified from a strain in which the only copy of infB (encoding for IF2) (RB419) was also under a P$_{spank}$ promoter. In the absence of IPTG the induced IF2 depletion causes the 70S ribosomes to dissociate into mature 50S and 30S subunits.*

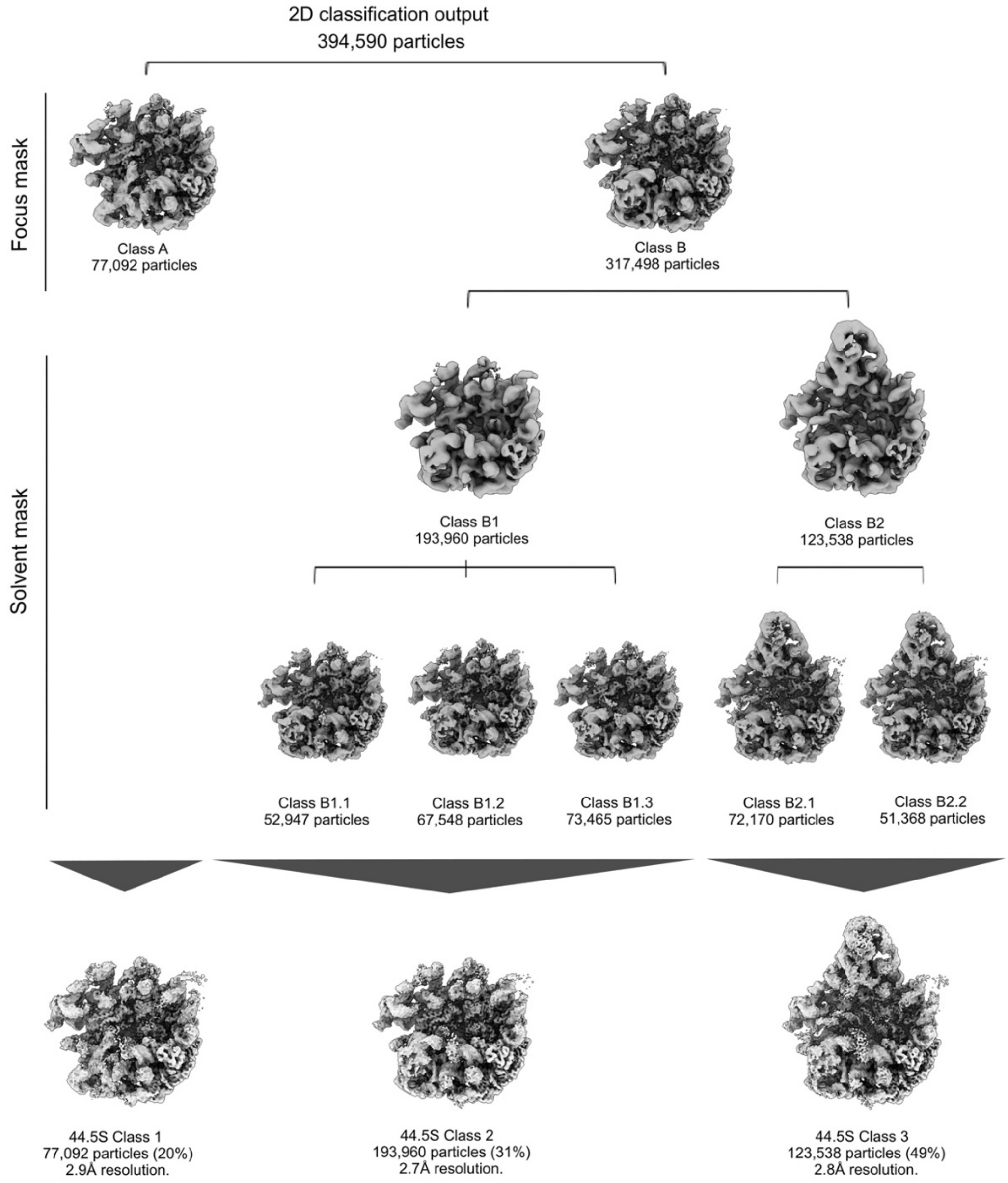

Supplementary Figure S2. ***3D classification and refinement workflow for the 44.5S$_{YsxC}$ particles isolated from YsxC-depleted cells.*** *The 44.5S$_{YsxC}$ particles were purified from the YsxC-depleted cells and imaged using cryo-EM. The particle images underwent an image classification pipeline as outlined in the diagram. This process included an initial classification step using a mask around the uL2 binding site, followed by a second classification step involving a solvent mask encompassing the entire particle. The second step comprised two layers of classification, where in each layer, particles were divided into two or three classes as indicated. The resulting subclasses were then clustered into three main classes. Particles within these three groups contributed to the production of the high-resolution cryo-EM maps depicted in Figure 1. The high-resolution cryo-EM maps are presented unsharpened. The number of particles employed for the final refinement of each class, along with the percentage of the total population they represent and the achieved resolution, is also indicated.*

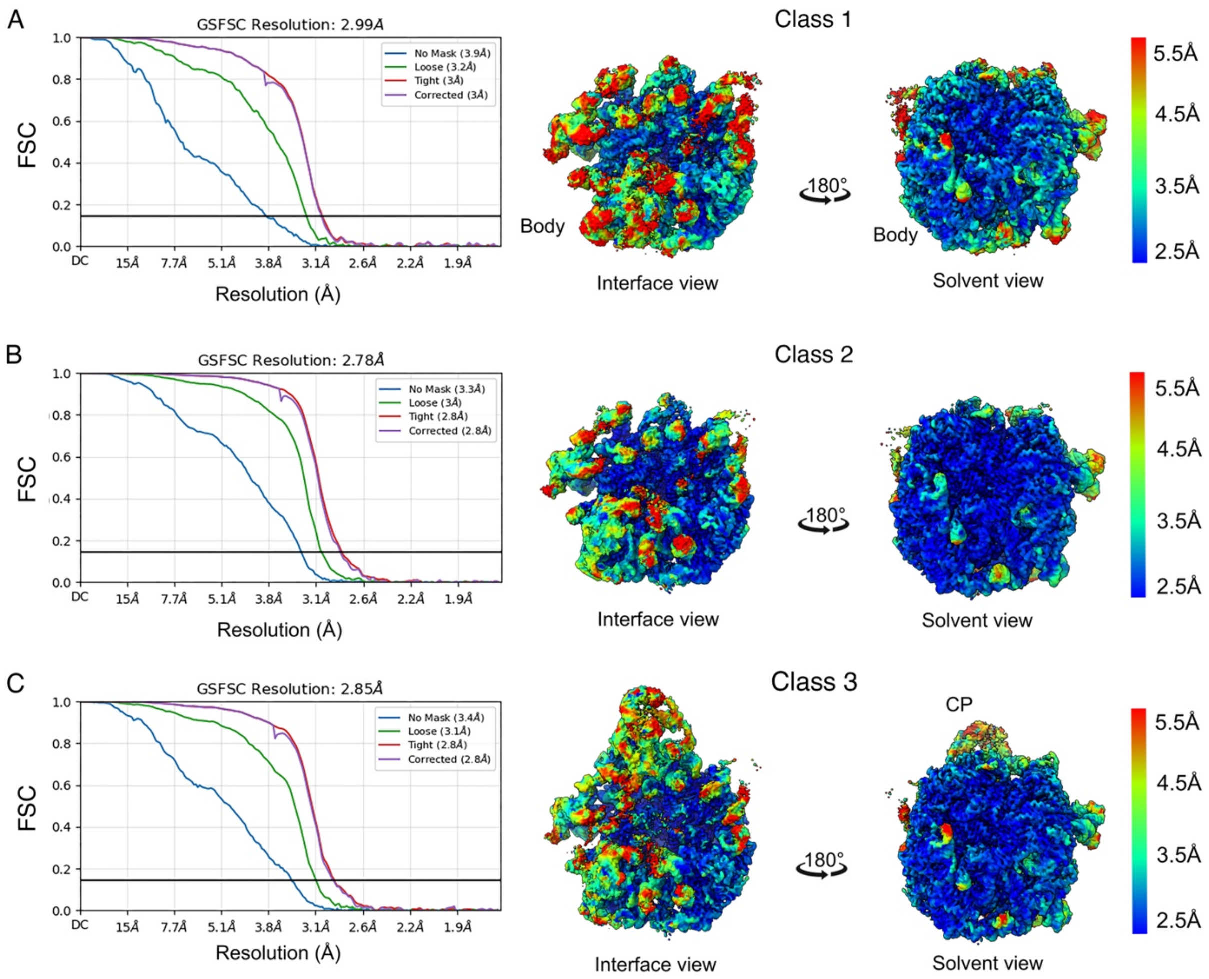

Supplementary Figure S3. ***Resolution analysis of the cryo-EM maps for the 44.5S$_{YsxC}$ particles isolated from YsxC cells.*** *(A-C) Gold-Standard Fourier Shell Correlation (GSFSC) plots (left) for the cryo-EM maps obtained from the three classes of 44.5S$_{YsxC}$ particles purified from YsxC-depleted cells. The FSC curves were calculated using different masks. In the 'No Mask' plot, the raw FSC curve was computed between two independent half-maps without any masking. 'Loose': FSC calculated after applying a loose soft solvent mask to both half-maps. The loose mask is determined by thresholding the density map at 50% of the maximum density value. The resulting volume is dilated to create a soft mask. Voxels in the mask within 25 Å of the thresholded region are assigned a mask value of 1.0. Voxels between 25 and 40 Å fall off with a soft cosine edge, while voxels outside 40 Å receive a value of 0.0. 'Tight' is similar to the 'Loose' mask, except the dilation distances are 6 Å for the value 1.0 and 12 Å for the value 0.0 distance. 'Corrected' uses the 'Tight' mask adjusted by noise substitution. In this case, the two half-maps have their phases randomized beyond a certain resolution, then the tight mask is applied to both, and a FSC plot is calculated. This FSC plot is used along with the original FSC plot before phase randomization to compute the corrected FSC plot, accounting for correlation effects induced by masking. The resolution at which phase randomization begins is the resolution where the no-mask FSC plot falls below the FSC = 0.143 criterion. The reported resolution is based on an FSC threshold of 0.143. The right panels show the front ('interface view') and back view ('solvent view') of the cryo-EM maps obtained for these classes, colored according to their local resolution. The local resolution scale bar and structural landmarks of the 50S subunit are indicated. CP signifies 'central protuberance'.*

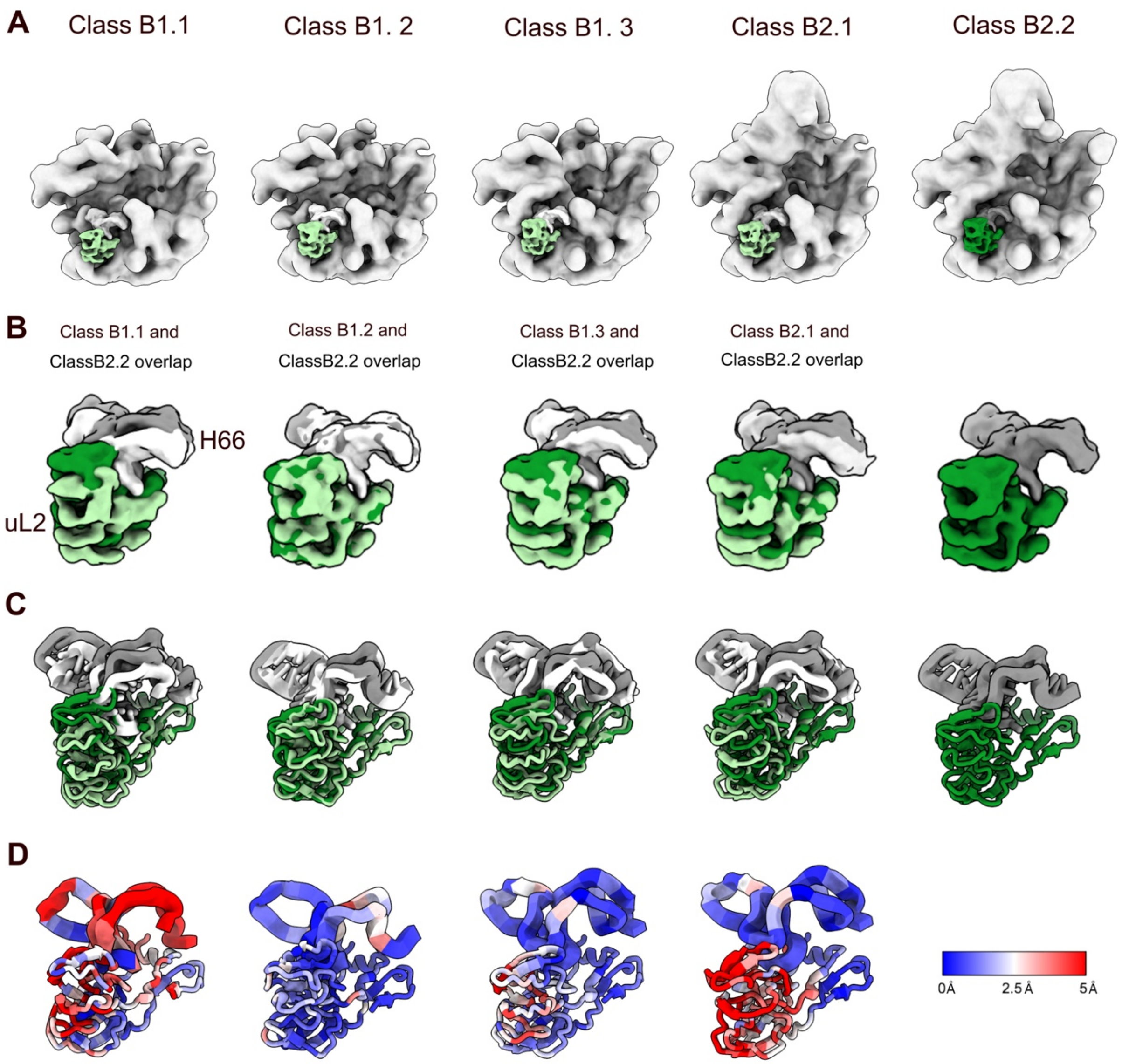

Supplementary Figure S4. ***Analysis of uL2 density in the 44.5S$_{YsxC}$ particles purified from the YsxC-depleted cells.*** *(A) Panels indicate the position of uL2 in the 44.5S$_{YsxC}$ particle classes purified from YsxC-depleted cells. uL2 is colored in light green for all classes except for class B2.2, which is shown as forest green. (B) The density associated with uL2 (in light green) and H66 (white) in each class was segmented out and overlapped with the uL2 density (in forest green) and H66 (grey) in class B2.2. (C) A molecular model was built from the uL2 and H66 densities in each class. The model from each class was overlapped with the model obtained for class B2.2. The color code in this panel is the same as in panel (B). (D) The molecular models built from the uL2 and H66 densities in each class were colored according to the root-mean-square deviation (r.m.s.d.) from the model obtained for class B2.2. The imperfect overlap of the densities and molecular models derived from the uL2 and H66 densities in the five classes from the 44.5S$_{YsxC}$ particle dataset indicates that uL2 binds to these particles in a wobbly manner.*

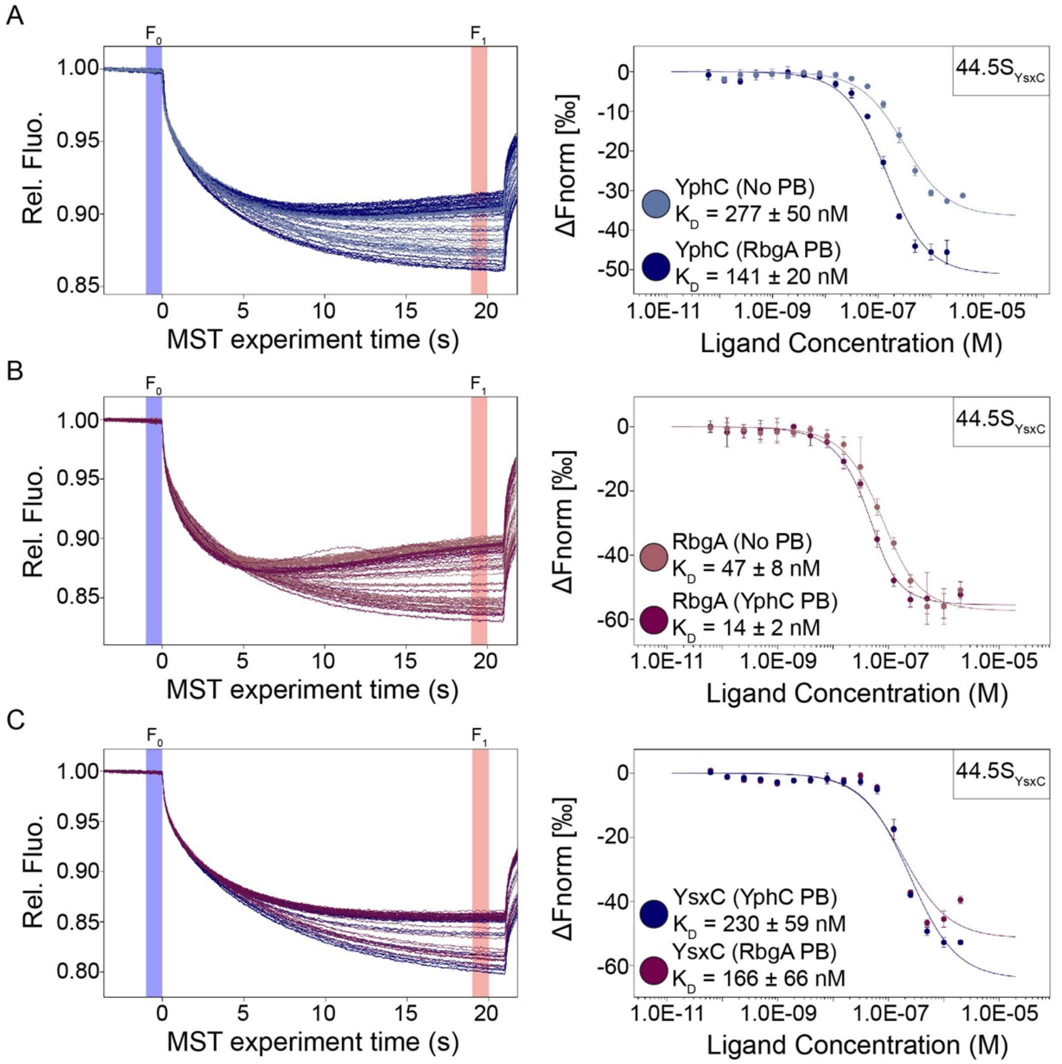

Supplementary Figure S5. ***Binding affinity of YphC and RbgA to the $44.5S_{YsxC}$ particle and effect of prebinding of YphC and RbgA in the binding of YsxC to the $44.5S_{YsxC}$ particle.*** *(A) Binding affinity measurement of YphC to the $44.5S_{YsxC}$ particle. This measurement was obtained by assembling the reaction with a constant 60 nM concentration of YphC and an increasing concentration of the ribosomal particle. To examine the effect of RbgA prebinding to the ribosomal particle on the binding affinity of YphC, RbgA was equimolar with the particle for each concentration tested. PB means 'prebound'. The left panel shows thermophoretic mobility traces of the MST reactions, depicting the individual traces for each ribosomal particle concentration and highlighting the F_0 (blue) and F_1 (red) regions used to calculate binding. The binding plots (right panel) depict ΔF_{norm} (F_1/F_0) versus particle concentration for the reaction measuring direct binding to the $44.5S_{YsxC}$ particle, or after prebinding of RbgA. The F_{norm} curves were fit using the law of mass action to derive K_d values. Dots represent the average from the three replicates at each concentration. Error bars denote standard deviation. (B) Binding affinity measurement of RbgA to the $44.5S_{YsxC}$ particle and the effect of YphC prebinding on RbgA binding affinity. (C) This panel shows the MST experiments to measure the impact of YphC and RbgA prebinding to the $44.5S_{YsxC}$ particle on the binding affinity of YsxC. Reactions in (B) and (C) were prepared as in (A), and the graphs are also presented using the same layout as in (A).*

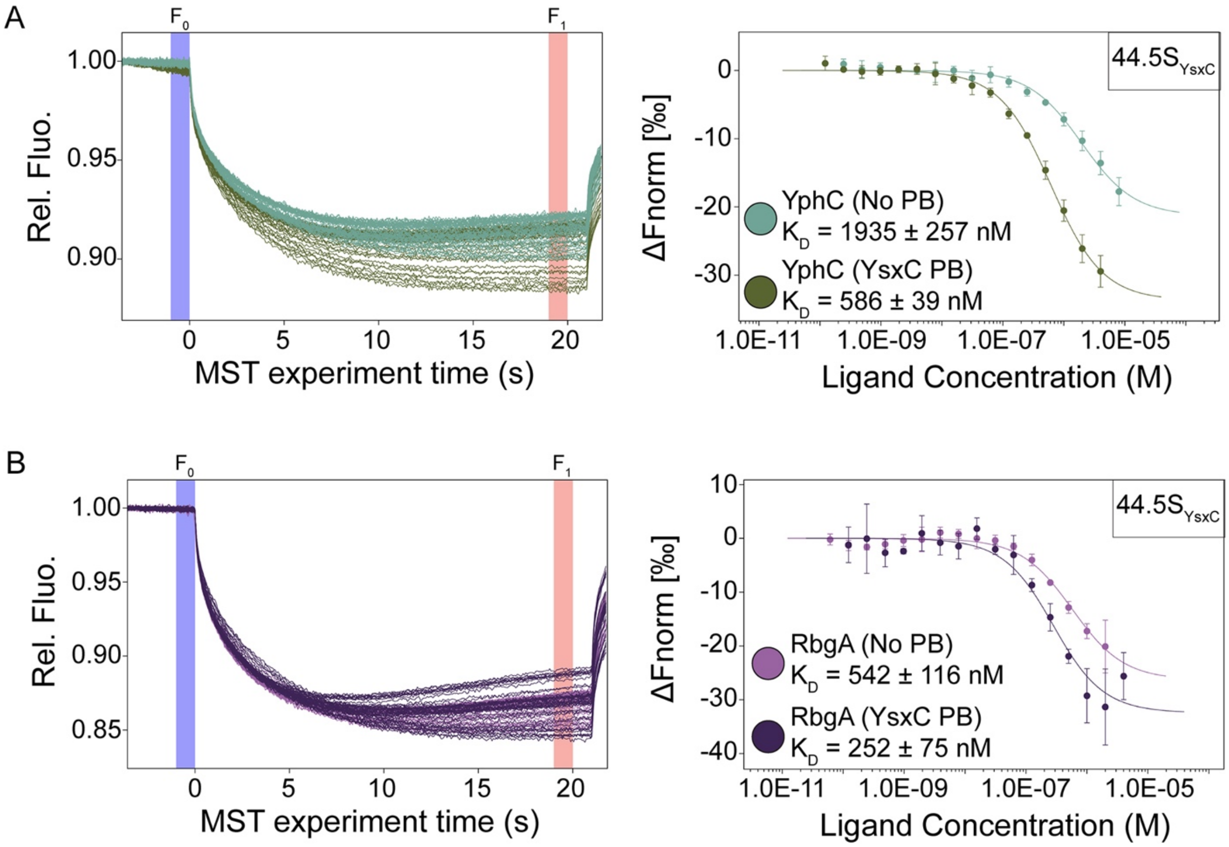

Supplementary Figure S6. ***Binding affinity of RbgA and YphC to the 44.5S$_{YsxC}$ particle and binding cooperativity between YphC, RbgA and YphC.*** *(A) Binding affinity measurement of YphC to the 44.5S$_{YsxC}$ particle. This measurement was obtained using the same experimental setup as in Supplementary Figure S5A and in the same MST buffer (see Materials and Methods section). However, this buffer contained a final concentration of 100 mM KCl. To examine the effect of YsxC prebinding to the ribosomal particle on the binding affinity of YphC, YsxC was equimolar with the particle for each concentration tested. PB means 'prebound'. The graphs are presented using the same layout as in Supplementary Figure S5. (B) Binding affinity measurement of RbgA to the 44.5S$_{YsxC}$ particle. This measurement was also obtained in MST buffer containing a final concentration of 100 mM KCl. To examine the effect of YsxC prebinding to the ribosomal particle on the binding affinity of RbgA, YsxC was equimolar with the particle for each concentration tested. The graphs are presented using the same layout as in Supplementary Figure S5.*

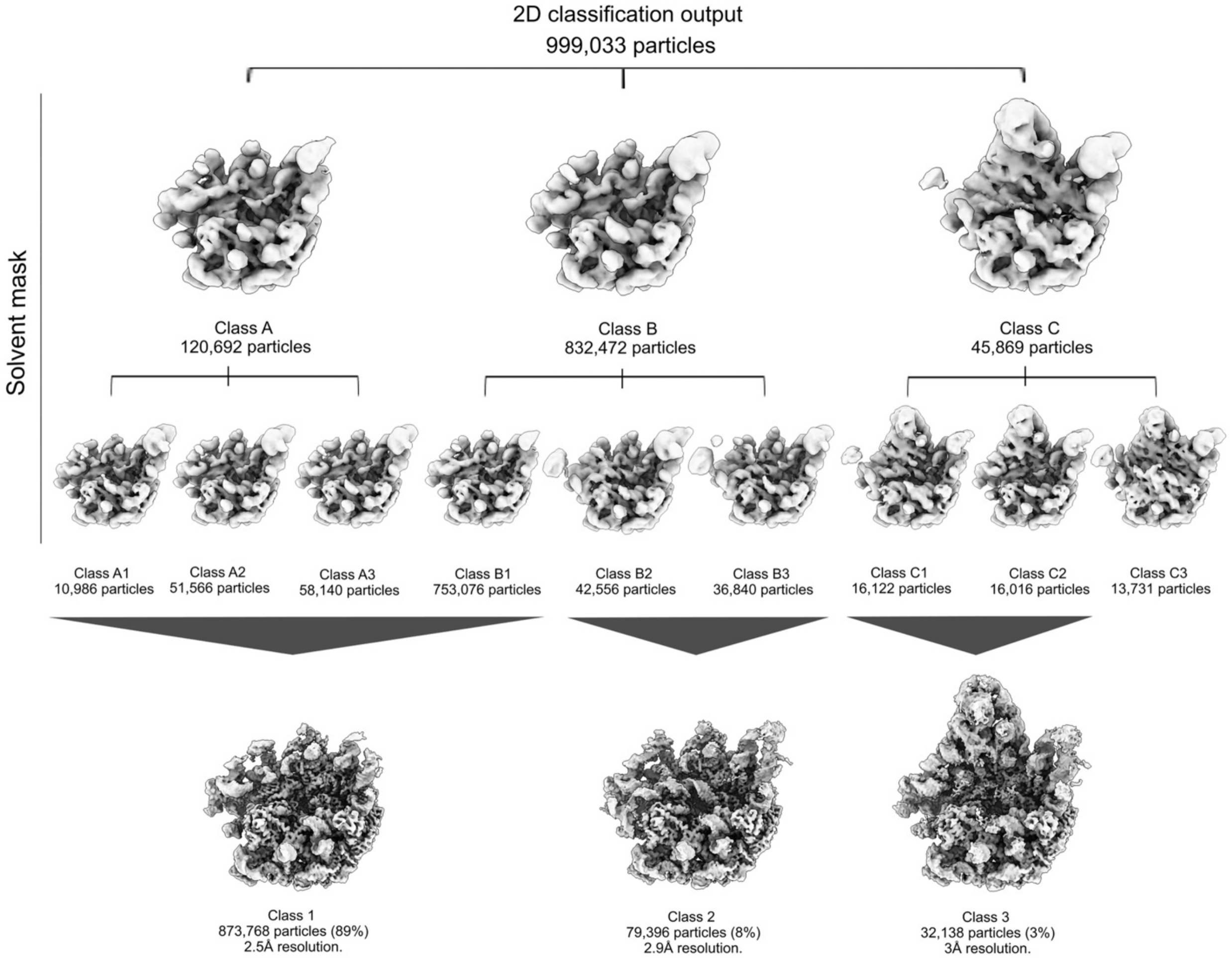

Supplementary Figure S7. ***3D classification and refinement workflow for the YsxC-GMPPNP treated 44.5S$_{YsxC}$ particles.*** *The 44.5S$_{YsxC}$ particles were purified from the YsxC-depleted cells and imaged by cryo-EM after incubation with YsxC at 37 °C for 15 minutes in the presence of a buffer containing 2 mM GMPPNP. The particle images were subjected to the image classification pipeline illustrated in the diagram. This classification included two layers of classification. In each layer, particles were divided into three classes as indicated. For these classifications, we used a solvent mask surrounding the entire particle. The resulting subclasses were organized into three main classes. Particles in these three groups were used to produce the high-resolution cryo-EM maps displayed in Figure 3. The high-resolution cryo-EM maps are presented unsharpened. The number of particles used for the final refinement of each class, the percentage of the total population they represent, and the obtained resolution are indicated.*

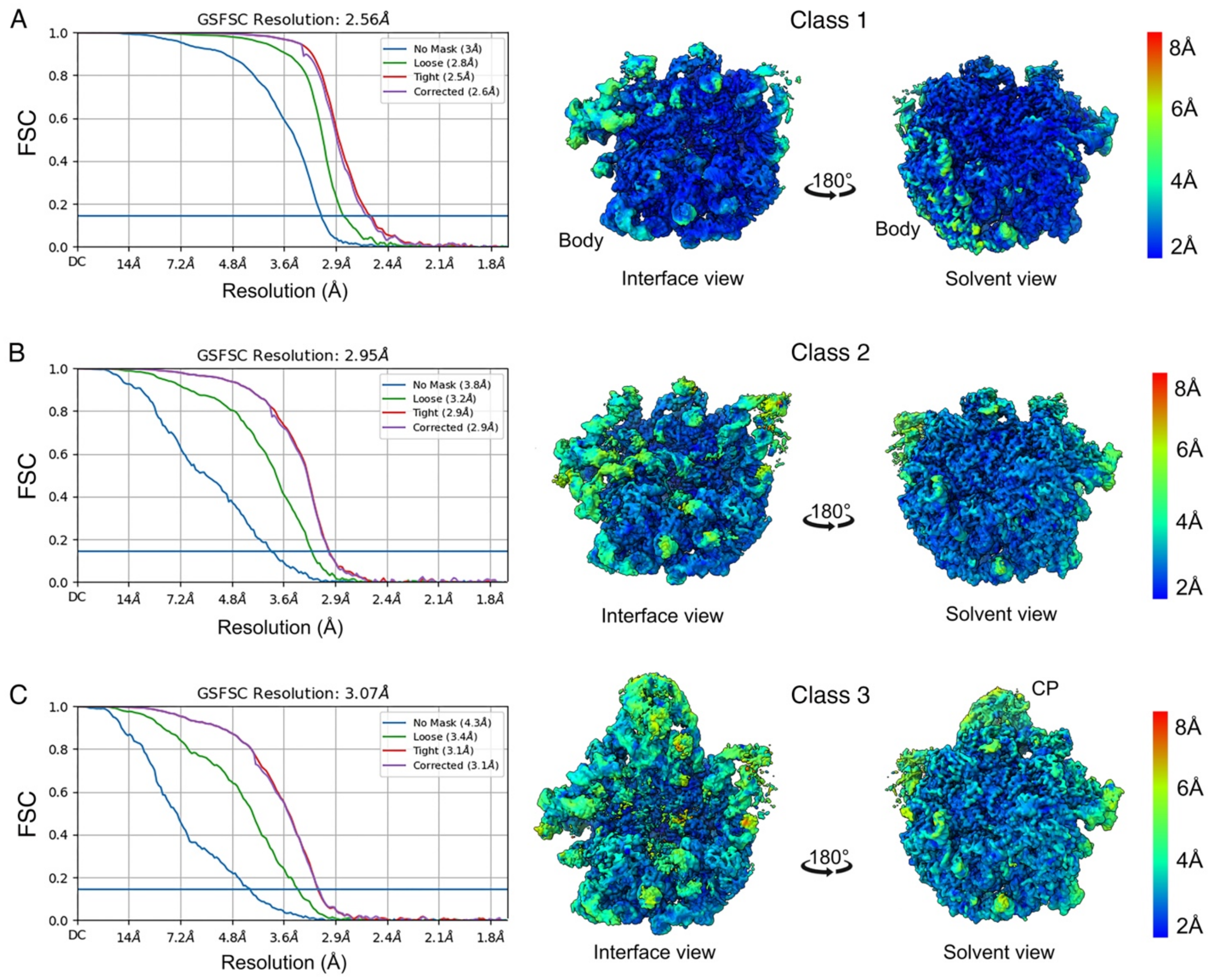

Supplementary Figure S8. ***Resolution analysis of the cryo-EM maps for the YsxC-GMPPNP treated 44.5S$_{YsxC}$ particles.*** *(A-C) Gold standard Fourier Shell Correlation (GSFSC) plots (left) for the cryo-EM maps obtained for the three classes of 44.5S$_{YsxC}$ particles after incubation with YsxC in a buffer containing 2 mM GMPPNP. The graphs on the left show the 'No Mask, ' 'Loose, '' Tight, 'and 'Corrected' FSC plots calculated as described in Supplementary Figure S1. The reported resolution is based on an FSC threshold of 0.143. The right panels show the front ('interface view') and back view ('solvent view') of the cryo-EM maps obtained for these classes, colored according to their local resolution. The local resolution scale bar and structural landmarks of the 50S subunit are indicated. CP stands for 'central protuberance. '*

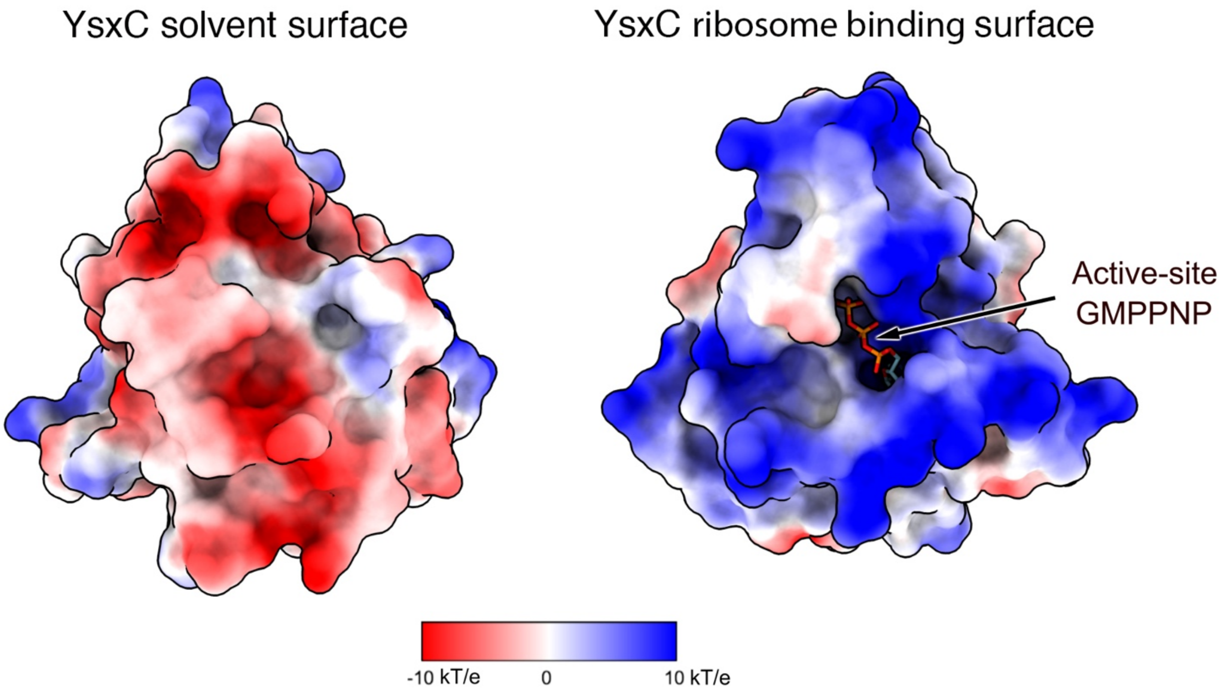

Supplementary Figure S9. *Electrostatic surface representation of YsXC.* *The panels illustrate the solvent and ribosome binding surfaces of YsxC, colored according to their electrostatic potential. These electrostatic surface representations of YsxC were derived from the molecular model of YsxC based on the cryo-EM maps for the YsxC-GMPPNP treated 44.5S_{YsxC} particles.*

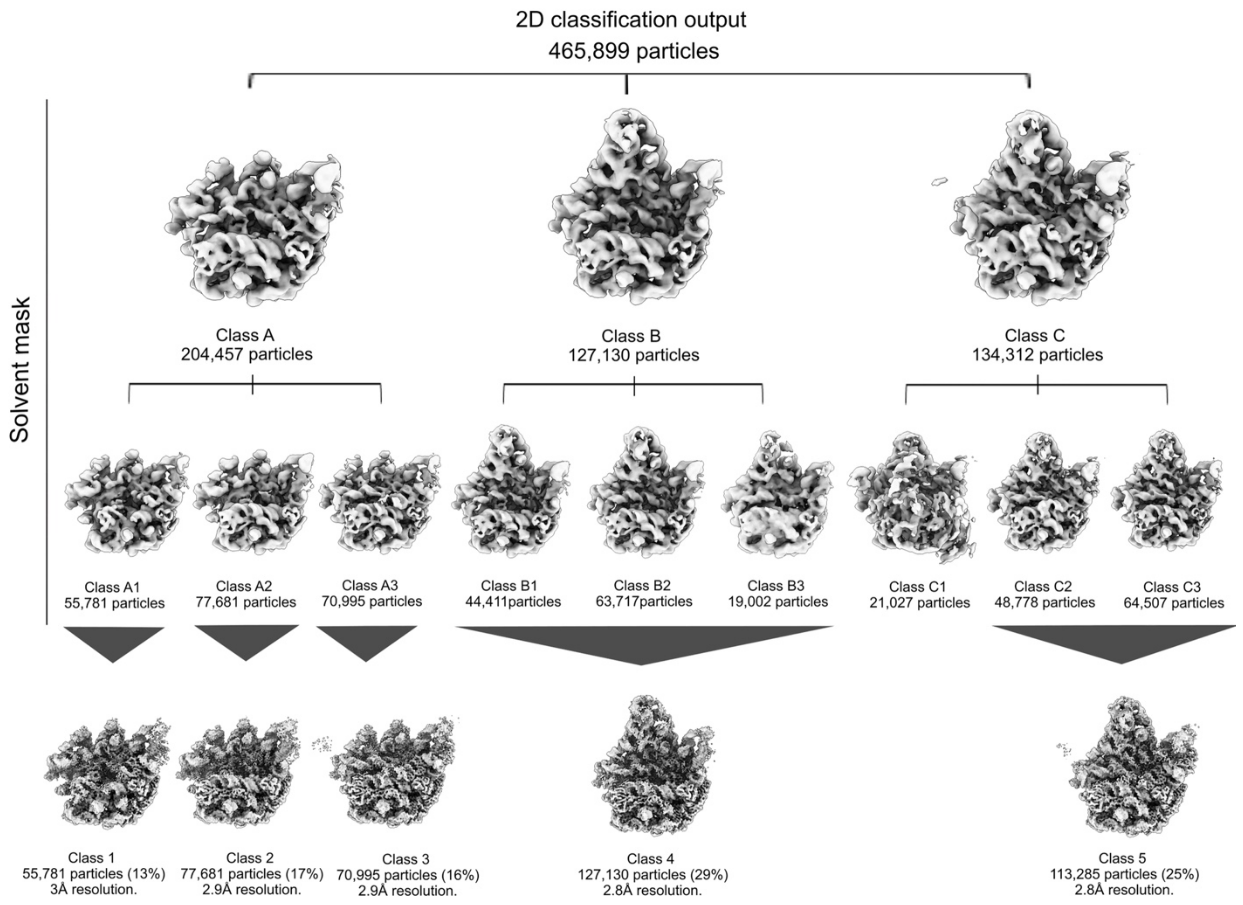

Supplementary Figure S10. ***3D classification and refinement workflow for the YsxC-GTP treated 44.5S$_{YsxC}$ particles.*** *The 44.5S$_{YsxC}$ particles were purified from YsxC-depleted cells and imaged by cryo-EM after incubation with YsxC at 37 °C for 15 minutes in the presence of a buffer containing 2 mM GTP. The particle images underwent the image classification pipeline outlined in the diagram. This classification involved two layers. In each layer, particles were divided into three classes as indicated. A solvent mask surrounding the entire particle was used for these classifications. The resulting subclasses were organized into five main classes. Particles in each group were utilized to generate the high-resolution cryo-EM maps displayed in Figure 6. The high-resolution cryo-EM maps are presented unsharpened. The number of particles used for the final refinement of each class, the percentage of the total population they represent, and the attained resolution are indicated.*

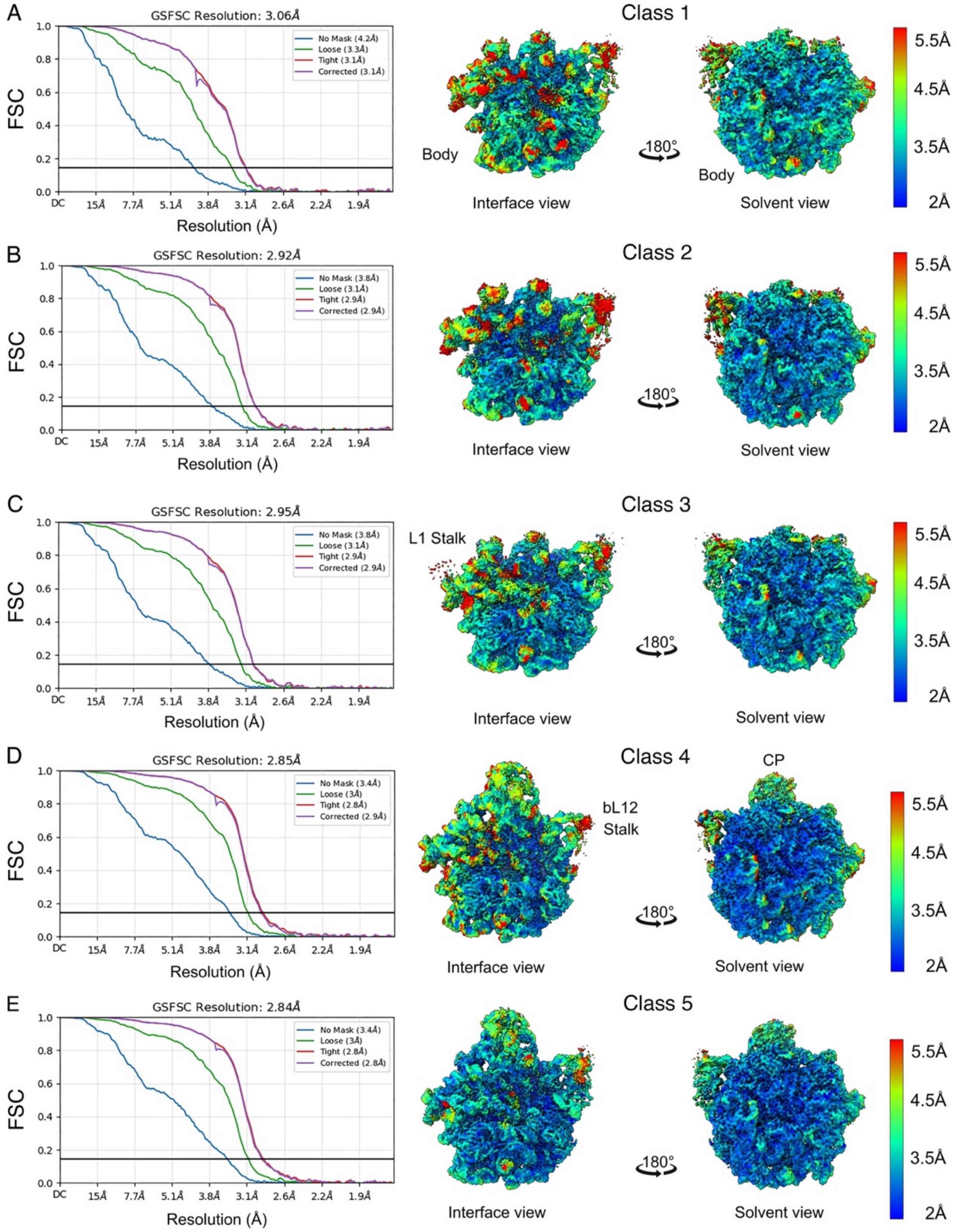

Supplementary Figure S11. ***Resolution analysis of the cryo-EM maps for the YsxC-GTP treated 44.5S$_{YsxC}$ particles.*** *(A-E) Gold-Standard Fourier Shell Correlation (GSFSC) plots (left) for the cryo-EM maps obtained from the five classes of 44.5S$_{YsxC}$ particles after incubation with YsxC in a buffer containing 2 mM GTP. The graphs on the left display the 'No Mask', 'Loose', 'Tight', and 'Corrected' FSC plots calculated as described in Supplementary Figure S1. The reported resolution is based on an FSC threshold of 0.143. The right panels exhibit the front view ('interface view') and back view ('solvent view') of the cryo-EM maps for these classes, colored according to their local resolution. A local resolution scale bar and the structural landmarks of the 50S subunit are indicated. CP refers to 'central protuberance'.*

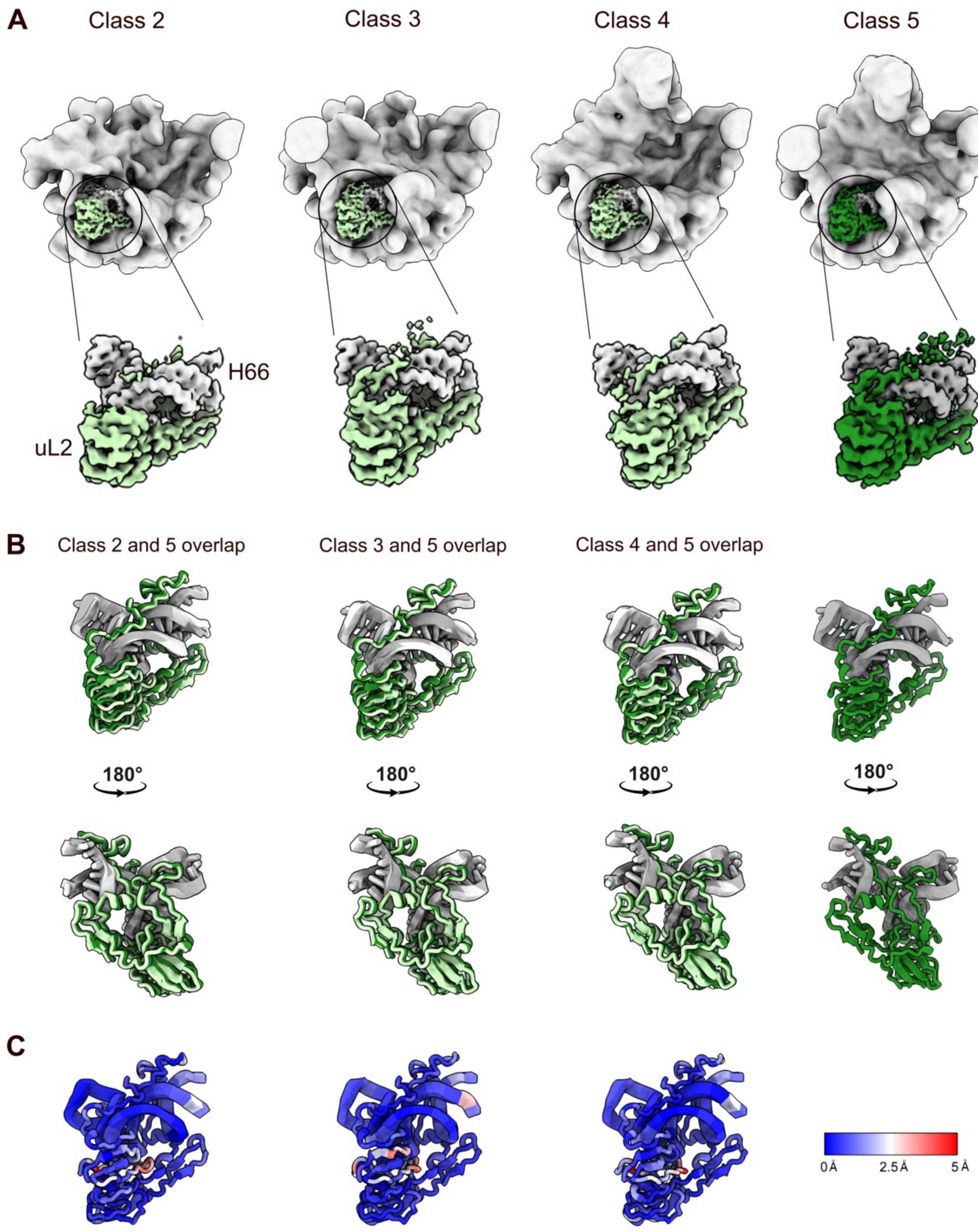

Supplementary Figure S12. ***Analysis of uL2 density in the YsxC-GTP treated 44.5S$_{YsxC}$ particles.*** *(A) Panels indicate the position of uL2 in the in the YsxC-GTP treated 44.5S$_{YsxC}$ particles. uL2 is colored in light green for all classes except for class 5, which is shown as forest green. (B) The density associated with uL2 (in light green) and H66 (white) in each class was segmented out and overlapped with the uL2 density (in forest green) and H66 (grey) in class 5. (C) A molecular model was built from the uL2 and H66 densities in each class. The model from each class was overlapped with the model obtained for class 5. The color code in this panel is the same as in panel (B). The top panels show the same views displayed in (A) and the bottom panels show the 180°rotated views with respect to the views in the top panels (C) The molecular models built from the uL2 and H66 densities in each class were colored according to the root-mean-square deviation (r.m.s.d.) from the model obtained for class 5. The close overlap of uL2 and H66 in all classes reveals that uL2 is stably bound.*

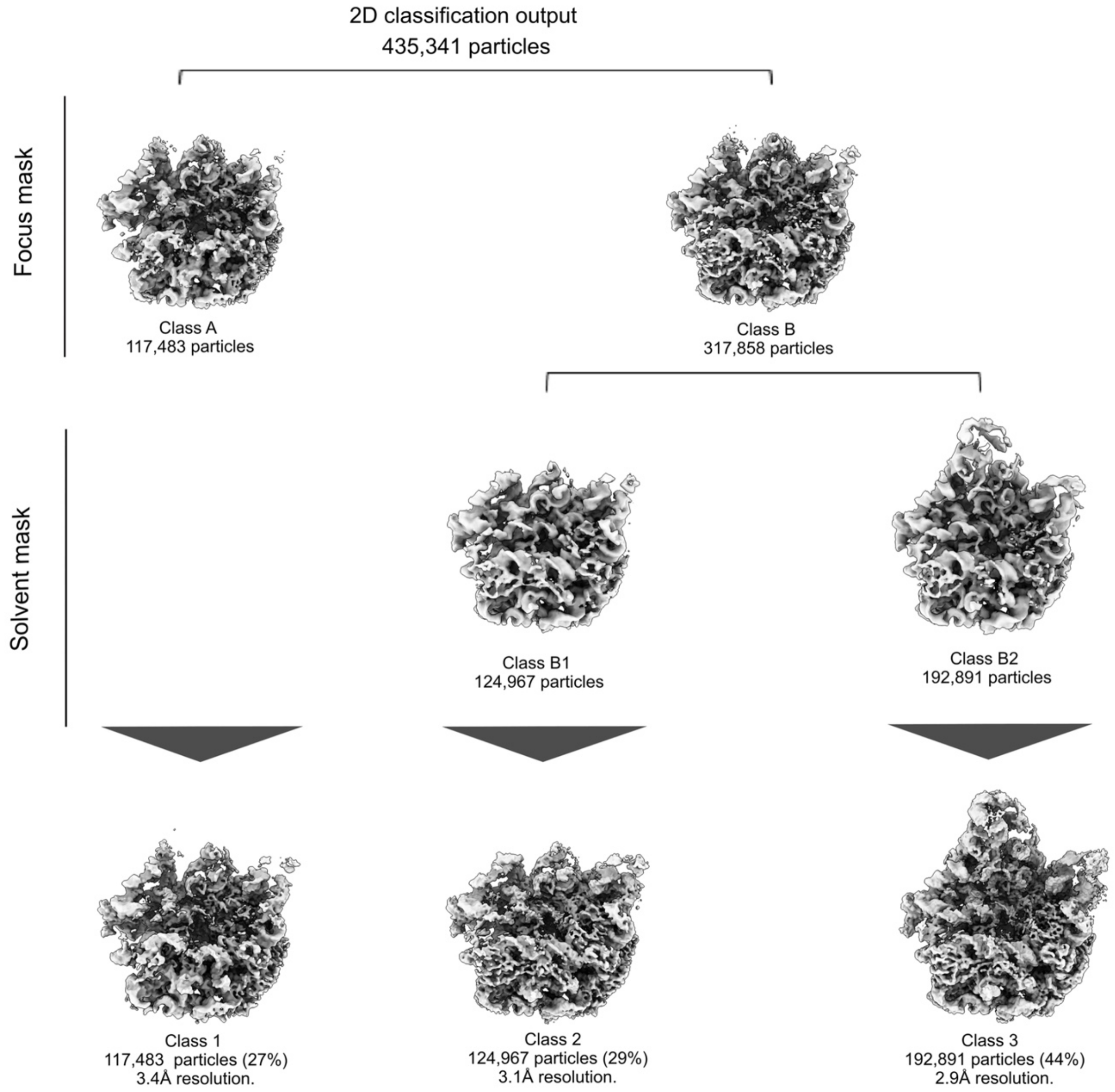

Supplementary Figure S13. ***3D classification and refinement workflow for the 44.5S$_{YsxC}$ particles after incubation.*** *The 44.5S$_{YsxC}$ Particles were purified from the YsxC-depleted cells and imaged by cryo-EM after incubation at 37 °C for 15 minutes. Particle images underwent the image classification pipeline described in the diagram. This included a first step of classification using a mask around the uL2 binding site and a second classification step using a solvent mask around the entire particle. The resulting subclasses were organized into three main classes. Particles in these three groups were utilized to produce the high-resolution cryo-EM maps shown in Figure 7. The high-resolution cryo-EM maps are presented unsharpened. The number of particles used for the final refinement of each class, the percentage of the total population they represent, and the obtained resolution are indicated.*

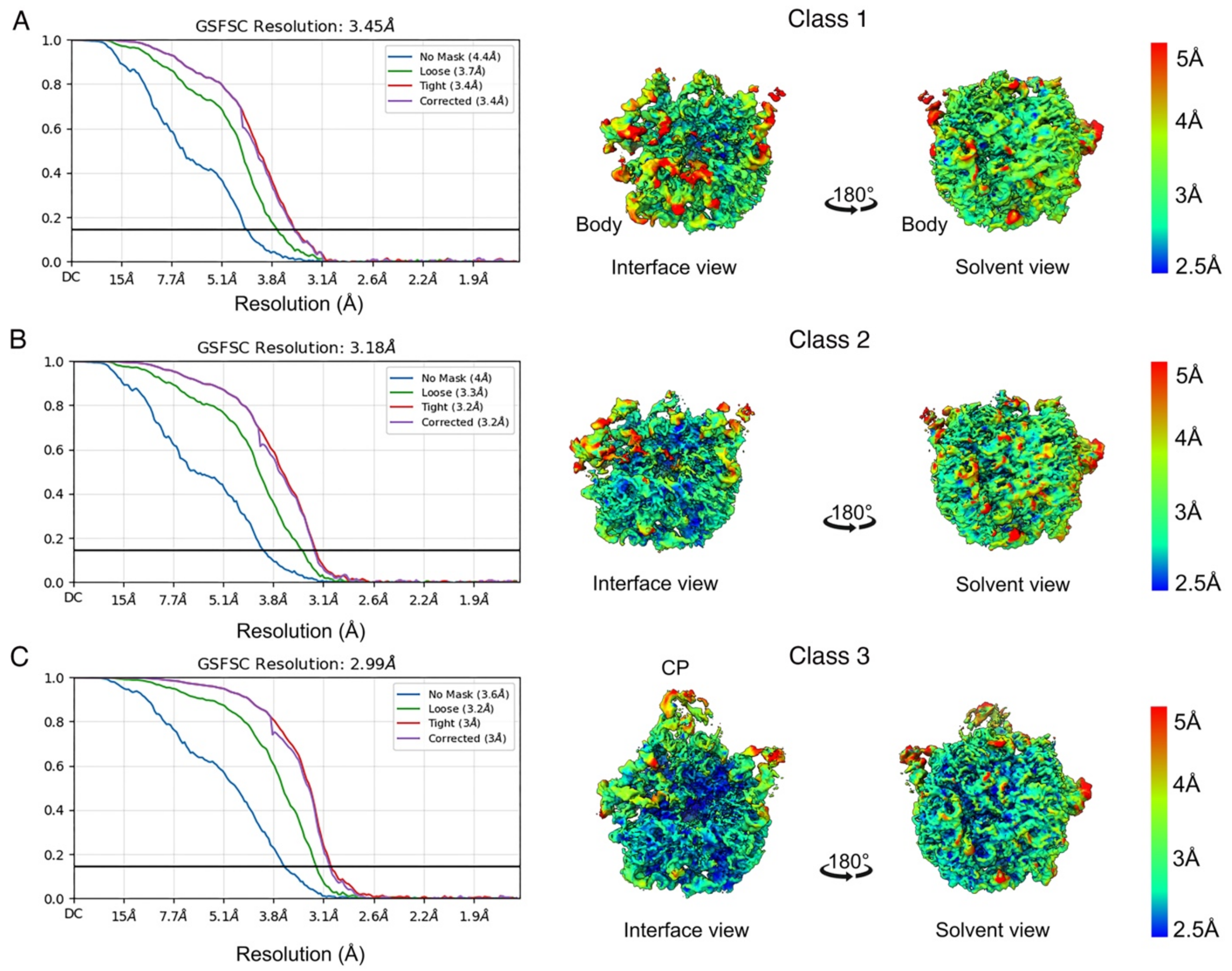

Supplementary Figure S14. ***Resolution analysis of the cryo-EM maps for the 44.5S$_{YsxC}$ particles after incubation.*** *(A-C) Gold-Standard Fourier Shell Correlation (GSFSC) plots (left) for the cryo-EM maps obtained for the three classes of 44.5S$_{YsxC}$ particles after incubation at 37 °C for 15 minutes. The graphs on the left display the 'No Mask', 'Loose', 'Tight', and 'Corrected' FSC plots calculated as described in Supplementary Figure S1. The reported resolution is based on an FSC threshold of 0.143. The right panels present the front ('interface view') and back view ('solvent view') of the cryo-EM maps obtained for these classes, colored according to their local resolution. A local resolution scale bar and structural landmarks of the 50S subunit are indicated. CP means 'central protuberance'.*

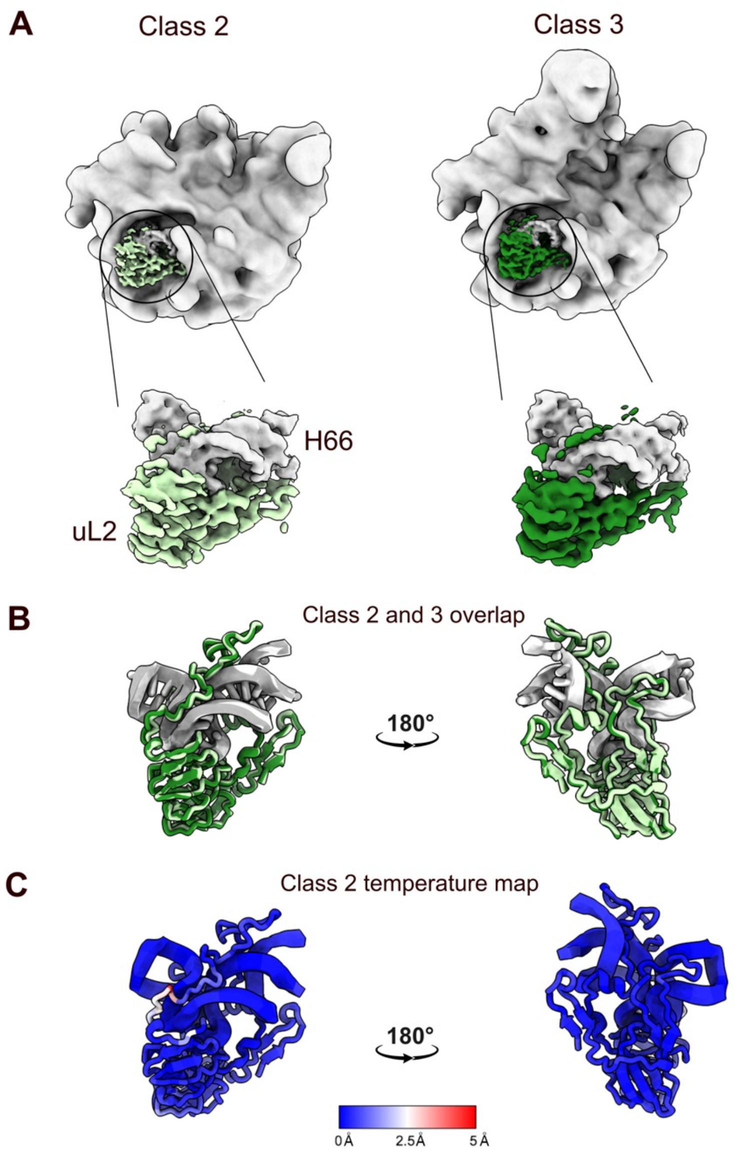

Supplementary Figure S15. ***Analysis of uL2 density in the 44.5S$_{YsxC}$ particles after incubation.*** *(A) Top panels indicate the position of uL2 in the 44.5S$_{YsxC}$ particles after incubation. uL2 is colored in light green for class 2 and in forest green for class 3. The density associated with uL2 and H66 were segmented out and shown in the bottom panels. The rRNA is colored in light grey. (C) A molecular model was built from the uL2 and H66 densities in each class, and the two models were overlapped. The color code in this panel is the same as in panel (B). The left panel show the same view displayed in (A) and the right panel show the 180°rotated view with respect to the left view. (C) The molecular model built from the uL2 and H66 densities in class 2 was colored according to the root-mean-square deviation (r.m.s.d.) from the model obtained for class 3. The r-protein uL2 and H66 in classes 2 and 3 overlapped relatively well, revealing that uL2 is more stably bound that in the 44.5S$_{YsxC}$ particles before incubation.*

SUPPLEMENTARY TABLES

Supplementary Table S1. ***Cryo-EM analysis of the 44.5S$_{YsxC}$ particles isolated from YsxC-depleted cells:*** *data acquisition parameters and map statistics.*

	44.5S$_{YsxC}$ particles isolated from YsxC-depleted cells		
	Class 1	Class 2	Class 3
**Data collection**			
**Microscope**	Titan Krios		
**Detector**	Gatan BioQuantum LS K3		
**Nominal Magnification**	105,000x		
**Voltage (kV)**	300		
**Total exposure ($e^-/Å^2$)**	50		
**Number of frames**	30		
**Defocus range (μm)**	-1.25 to -2.75		
**Calibrated physical pixel size (Å/px)**	0.855		
**Reconstruction and refinement**			
**Particles**	77,092	193,960	123,538
**Resolution (Å)**	2.9	2.7	2.8
**Data Deposition**			
**EMDB code**	71096	71099	71098

Supplementary Table S2. ***Cryo-EM analysis of the YsxC-GMPPNP treated 44.5S$_{YsxC}$ particles:*** *data acquisition parameters, map statistics and molecular model validation parameters.*

		YsxC-GMPPNP treated 44.5S$_{YsxC}$ particles		
		Class 1	Class 2	Class 3
**Data collection**				
**Microscope**		Titan Krios		
**Detector**		Gatan BioQuantum LS K3		
**Nominal Magnification**		105,000x		
**Voltage (kV)**		300		
**Total exposure (e$^-$/Å^2)**		40		
**Number of frames**		30		
**Defocus range (µm)**		-1.25 to -2.75		
**Calibrated physical pixel size (Å/px)**		0.855		
**Reconstruction and refinement**				
**Particles**		873,768	79,396	32,138
**Resolution (Å)**		2.5	2.9	3
**Model composition**				
**RNA chains**		1	-	-
**Protein chains**		17	-	-
**Model Building**				
**Protein Geometry**	Poor rotamers	0.50%	-	-
	Favored rotamers	81.16%	-	-
	Ramachandran outliers	0.32%	-	-
	Ramachandran favored	92.77%	-	-
	Rama distribution Z-score	-1.57 ± 0.18	-	-
	Cβ deviations >0.25Å	0.06%	-	-
	MolProbity score	1.98	-	-
	Bad bonds	0%	-	-
	Bad angles	0.08%	-	-
	Cis-Proline	0%	-	-
**Nucleic Acid Geometry**	Probably wrong sugar puckers	2.27%	-	-
	Bad backbone conformations	27.36%	-	-
	Bad bonds	0%	-	-
	Bad angles	0.01%	-	-
**Low-resolution criteria**	CaBLAM outliers	3.7%	-	-
	CA Geometry outliers	0.87%	-	-
**Additional validations**	All-atom clash score	9.93	-	-
**Data Deposition**				
EMDB / PDB codes		71238/ 9P38	71102	71103

Supplementary Table S3. ***Cryo-EM analysis of the YsxC-GTP treated 44.5S$_{YsxC}$ particles:*** *data acquisition parameters, map statistics and molecular model validation parameters*

		YsxC-GTP treated 44.5S$_{YsxC}$ particles				
		Class 1	Class 2	Class 3	Class 4	Class 5
**Data collection**						
**Microscope**		Titan Krios				
**Detector**		Gatan BioQuantum LS K3				
**Nominal Magnification**		105,000x				
**Voltage (kV)**		300				
**Total exposure (e$^-$/Å^2)**		50				
**Number of frames**		30				
**Defocus range (µm)**		-1.25 to -2.5				
**Calibrated physical pixel size (Å/px)**		0.855				
**Reconstruction and refinement**						
**Particles (used in final refinement step)**		55,781	77,681	70,995	127,130	113,285
**Resolution (Å)**		3	2.9	2.9	2.8	2.8
**Model composition**						
**RNA chains**		-	-	-	-	2
**Protein chains**		-	-	-	-	21
**Model Building**						
**Protein Geometry**	Poor rotamers	-	-	-	-	0.54%
	Favored rotamers	-	-	-	-	71.73%
	Ramachandran outliers	-	-	-	-	0.08%
	Ramachandran favored	-	-	-	-	88.24%
	Rama distribution Z-score	-	-	-	-	-2.65 ± 0.16
	Cβ deviations >0.25Å	-	-	-	-	0%
	MolProbity score	-	-	-	-	2.17
	Bad bonds	-	-	-	-	0%
	Bad angles	-	-	-	-	0.08%
	Cis-Proline	-	-	-	-	1.08%
**Nucleic Acid Geometry**	Probably wrong sugar puckers	-	-	-	-	2.66%
	Bad backbone conformations	-	-	-	-	38.94%
	Bad bonds	-	-	-	-	0%
	Bad angles	-	-	-	-	0.03%
**Low-resolution criteria**	CaBLAM outliers	-	-	-	-	6.5%
	CA Geometry outliers	-	-	-	-	1.63%
**Additional validations**	All-atom clash score	-	-	-	-	11.40
**Data Deposition**						
EMDB / PDB codes		71104	71105	71106	71107	71268/ 9P4I

Supplementary Table S4. ***Cryo-EM analysis of the 44.5S$_{YsxC}$ particles after incubation at 37 °C for 15 min:*** *data acquisition parameters and map statistics.*

	44.5S$_{YsxC}$ particles *after incubation at 37 °C for 15 min*		
	Class 1	Class 2	Class 3
**Data collection**			
**Microscope**	Titan Krios		
**Detector**	Gatan BioQuantum LS K3		
**Nominal Magnification**	105,000x		
**Voltage (kV)**	300		
**Total exposure (e$^-$/Å^2)**	50		
**Number of frames**	30		
**Defocus range (µm)**	-1.25 to -2.75		
**Calibrated physical pixel size (Å/px)**	0.855		
**Reconstruction and refinement**			
**Particles**	117,483	124,967	192,891
**Resolution (Å)**	3.4	3.1	2.9
**Data Deposition**			
**EMDB code**	71109	71110	71111
